# Calibrating microglia states in Alzheimer’s disease: decoding immune-metabolic networks and nano-targeted multicomponent therapies

**DOI:** 10.3389/fimmu.2026.1843978

**Published:** 2026-06-16

**Authors:** Jin Feng Xing, Kaijin Mu, Xue Yan, Xu Yang, Dongnan Zhang, Wanning Gao, Tengyue Zhang, Shuangying Yang, Runze Wang, Weimin Zhang, Yilong Zhu

**Affiliations:** 1 School of Integrated Traditional Chinese and Western Medicine, Changchun University of Chinese Medicine, Changchun, China; 2 Brain Disease Center, Rehabilitation Department, The Third Affiliated Clinical Hospital of Changchun University of Chinese Medicine, Changchun, China; 3 Brain Disease Center, The Third Affiliated Clinical Hospital of Changchun University of Chinese Medicine, Changchun, China; 4 College of Basic Medical Sciences, Changchun University of Chinese Medicine, Changchun, China; 5 School of Rehabilitation Medicine, Changchun University of Chinese Medicine, Changchun, China; 6 Key Laboratory of Jilin Province for Traditional Chinese Medicine Prevention and Treatment of Infectious Diseases, College of Integrative Medicine, Changchun University of Chinese Medicine, Changchun, China

**Keywords:** Alzheimer’s disease, APOE ϵ4, complement cascade, immunometabolism, microglia, nanomedicine, TREM2

## Abstract

Alzheimer’s disease treatment is shifting from pathology removal to regulating the brain microenvironment. Anti-Aβ monoclonal antibodies, such as lecanemab and donanemab, provide statistically significant disease-modifying effects but offer only modest cognitive improvement and pose safety risks, including amyloid-related imaging abnormalities. These results show that amyloid clearance is clinically relevant but not sufficient for full restoration of neuroimmune, metabolic, synaptic, and neurovascular balance. Microglia are now seen as central to Alzheimer’s disease susceptibility and progression, existing along dynamic, spatially organized, sex-influenced, and genetically determined continua beyond a simple pro- or anti-inflammatory state. This review calls out three key drivers of microglial dysfunction: the TREM2–APOE lipid-sensing axis, complement-mediated synaptic elimination, and immunometabolic reprogramming—including glycolysis, mitochondrial damage, autophagy failure, NAD+ depletion, and innate immune signaling. We examine natural bioactive compounds, metabolic modulators, and biomimetic nanodelivery as promising, yet currently unproven, strategies for adjusting microglial state. Future therapies should incorporate both pathology removal and microenvironment protection, tailored by disease stage, genetic profile, sex, vascular risk, and microglial state-associated biomarkers.

## 1 Introduction

Alzheimer’s disease (AD) is an urgent public health challenge as the global population ages (1, 2). Disease-modifying therapies that slow or halt progression are now critical (3, 4). For over three decades, Alzheimer’s drug development has focused on the amyloid cascade hypothesis, which asserts that abnormal amyloid-beta drives downstream tau pathology and cognitive decline (5). Despite important advances, targeting amyloid alone has not consistently produced sustained clinical benefits (3, 4, 6, 7).

Recent phase III trials of anti-Aβ monoclonal antibodies have decisively changed the field. Lecanemab and donanemab show that amyloid lowering achieves statistically significant disease modification in early symptomatic AD (8, 9), marking a clear clinical milestone after decades of failed trials (3, 4, 6). Nonetheless, cognitive benefits remain limited, and adverse events, such as amyloid-related imaging abnormalities, require careful monitoring (8–10). These findings confirm that amyloid drives pathology but underscore that it operates within a broader, complex network including tau, synaptic degeneration, vascular dysfunction, innate immunity, lipid metabolism, and cellular senescence (10–13).

Genomic studies firmly reinforce this systems-level view. Genome-wide association studies and exome sequencing establish that key late-onset AD risk genes—including *TREM2*, *CD33*, *MS4A6A*, *CR1*, *PLCG2*, *INPP5D*, and *ABCA7*—are enriched in microglia, not just vulnerable neurons (14–20). Microglia must now be recognized as active determinants of disease susceptibility and progression, not passive responders (11, 14, 19, 20). The dual regulatory roles of *MS4A6A* and related modules solidify microglia as upstream immune-sensing hubs (21).

Single-cell transcriptomics and spatial omics demand a rethinking of microglial states. The M1/M2 framework is inadequate. In AD, microglia occupy continuous, dynamic state spaces shaped by plaque proximity, disease stage, genotype, age, sex, and tissue context (22–30). Biological sex is crucial, directly affecting microglial inflammatory thresholds, lipid metabolism, complement activity, and vulnerability to senescence-like states, especially via sex-by-genotype interactions involving *APOE* ϵ4 (29–31).

This review asserts that future Alzheimer’s therapies must shift from single-target approaches to microglial state-calibration (12, 13, 31). We address three mechanistic axes: the TREM2–APOE lipid-sensing axis (32), complement-mediated synaptic elimination (33–36), and immunometabolic reprogramming involving glycolysis, mitochondrial dysfunction, autophagy failure, NAD+ depletion, and innate immune signaling (37–41). We evaluate network pharmacology, natural bioactive molecules, metabolic modulators, nanodelivery systems, and antibody-adjunctive strategies as promising approaches for clearing pathology and protecting the microenvironment (12, 13, 42–44).

## 2 Neuroimmune imbalance and epigenetic memory in microglia in AD

Microglia have a unique developmental origin and lifespan. Unlike peripheral macrophages, they arise from yolk-sac erythroid-myeloid progenitors, enter the embryonic brain, and self-renew locally throughout life (45, 46). After formation of the blood-brain barrier, microglia remain largely separated from the hematopoietic system (46, 47). Their long residence in the brain means that aging, metabolic stress, inflammatory exposure, vascular injury, and protein aggregates can leave cumulative molecular marks on these cells (22, 47–49). The conceptual evolution of microglial research in Alzheimer’s disease has progressed from early morphological observations to modern single-cell spatial multi-omics (**Table 1**).

**Table 1:** Chronological milestones in the evolving understanding of microglia in Alzheimer’s disease.

Chronological milestones in the evolving understanding of microglia in Alzheimer’s disease				
Phase	Year	Key milestone	Lead author	Reference(s)
Foundational period (1918–1980)	1919	Microglia were first identified as a distinct resident immune cell population in the central nervous system using silver carbonate staining and were formally named “microglia.”	Pio del Rio-Hortega	(50)
	1921	Microglial morphological and functional activation after central nervous system injury was described, establishing the concept of microglia as intrinsic immune effector cells.	Pio del Rio-Hortega	(51)
	1925	Microglial phagocytic activity was documented in human gliomas.	Wilder Penfield	(52)
	1932	The first systematic English-language account of microglia was published.	Pio del Rio-Hortega	(53)
Functional exploration (1981–2010)	1987	Reactive HLA-DR-positive microglia were demonstrated in Alzheimer’s disease brains, firmly linking neuroinflammation to disease pathology.	McGeer PL	(54)
	1992	Amyloid-beta was shown to activate the classical complement cascade directly, connecting complement signaling to Alzheimer’s disease pathology.	Rogers J	(55)
	1994	APOE isoform-dependent differences in amyloid-beta binding affinity were demonstrated under non-denaturing, lipid-associated conditions.	LaDu MJ	(56)
	1998	The CX3CL1-*CX3CR1* axis was identified as a regulator of neuron-microglia communication.	Harrison JK	(57)
	2001	A subset of non-steroidal anti-inflammatory drugs was found to reduce amyloidogenic Abeta42, suggesting a new route for modulating amyloid generation.	Weggen S	(7)
	2007	Genetic association analyses linked multiple Alzheimer’s disease susceptibility loci to immune pathways.	Bertram L	(17)
	2010	Lineage-tracing studies demonstrated that adult microglia originate from embryonic yolk-sac progenitors.	Ginhoux F	(45)
Mechanistic and translational era (2011–2025)	2013	Exome sequencing showed that loss-of-function *TREM2* variants markedly increase Alzheimer’s disease risk.	Guerreiro R; Jonsson T	(15, 18)
	2016	Complement-dependent microglial synaptic pruning was identified as a core mechanism of early synapse loss in Alzheimer’s disease models.	Hong S	(33)
	2017	Single-cell RNA sequencing first defined disease-associated microglia (DAM) in Alzheimer’s disease models.	Keren-Shaul H; Krasemann S	(58, 59)
	2020	Spatial transcriptomics revealed spatial heterogeneity of microglial states in Alzheimer’s disease tissue.	Chen WT	(25)
	2023	Histone lactylation was shown to drive pro-inflammatory state transitions in senescent microglia.	Wei L	(60)
	2024	Exhausted-like microglial populations in aged and *APOE*4 Alzheimer’s disease brains were characterized at transcriptomic and functional levels.	Millet A	(29)

The table summarizes conceptual milestones that shaped current views of microglial biology in Alzheimer’s disease. Reference numbers correspond to the manuscript bibliography. AD, Alzheimer’s disease; APOE, apolipoprotein E; DAM, disease-associated microglia.

In AD, Aβ oligomers, plaque-associated lipids, tau-associated injury, vascular stress, and damage-associated molecular patterns released by injured neurons can activate microglia through pattern-recognition and lipid-sensing receptors (11, 32, 61, 62). Chronic stimulation of these receptors disrupts NF-κB signaling, leading to increased release of TNF-α, IL-1β, reactive oxygen species, and other neurotoxic mediators, while reducing the efficiency of phagocytic clearance (11, 61–64). This disruption creates an inflammatory environment that accelerates tau hyperphosphorylation and trans-synaptic tau propagation, thereby establishing a self-reinforcing neurodegenerative loop (11, 61, 62). The lack of reliable clinical success with traditional broad anti-inflammatory strategies, such as nonsteroidal anti-inflammatory drugs (6, 7), indicates that broad suppression of inflammation does not interrupt these mechanisms, and that more state-specific immune recalibration may be required (12, 13, 31).

### 2.1 Beyond M1/M2: unstable intermediate microglial states

The M1/M2 polarization model does not work for AD. Microglial activation is not simply a switch from “resting” to “inflammatory” or “harmful” to “protective.” Microglia often pass through unstable intermediate states (22–24, 65, 66). As they shift from homeostatic to disease-associated, neurodegenerative, interferon-responsive, or exhausted phenotypes, they may express homeostatic markers such as *P2RY12* or *TMEM119*, as well as activation-associated genes such as *APOE*, *LPL*, *CLEC7A*, *CST7*, *SPP1*, and interferon-stimulated genes (22, 25, 27–29, 58, 59).

This mixed transcriptional identity should not be dismissed as technical noise. It may reflect biological plasticity, asynchronous signaling, regional niche effects, and incomplete commitment to a disease-associated program (22–28). Recent studies support that microglial state transitions are dynamic and partially reversible in some contexts. They are shaped by metabolic, vascular, and inflammatory cues rather than by fixed lineage switches (37–40, 65, 66). Signaling at the neurovascular interface, including PI3K/AKT/mTOR, helps shape intermediate microglial polarization states. This reinforces the concept that microglial phenotypic transitions are context-dependent and can be modified (67). Therapeutically, targeting intermediate-state microglia in the “gray zone”—rather than exhausted or senescent cells—may increase the likelihood of effective recalibration and disease intervention (28, 29, 31).

### 2.2 Epigenetic memory: persistence, dilution, and therapeutic opportunity

Aging and chronic pathological conditions alter the microglial epigenome through changes in chromatin accessibility, DNA methylation, histone acetylation and methylation, and metabolic-epigenetic modifications, such as histone lactylation (49, 60, 68). Histone acetylation changes occur in human AD brain tissue, linked to aging, tau pathology, and neuroinflammatory gene regulation (68). Evidence for H3K18 lactylation as a driver of senescent microglial inflammation is mainly from experiments using senescent microglial cells and AD mouse models (60).

H3K18la is a metabolically linked, reversible chromatin modification. It is not a permanent epigenetic memory in human AD microglia (49, 60). Microglia live long but renew slowly (48), so some chromatin marks gained from stress may dilute during cell division or change as transcription factor occupancy and metabolic substrate availability shift (48, 49).

This reversibility offers a therapeutic opportunity. Chromatin marks, such as H3K18 lactylation, are dynamically maintained by the ongoing activity of specific “writers,” “readers,” and “erasers.” Targeting these enzymes, or the metabolites (lactate, acetyl-CoA, NAD+) that fuel them, could reset the microglial inflammatory threshold rather than erase a fixed scar (49, 60, 68). In this framework, epigenetic memory is not a permanent record of past pathology, but a metabolically coupled and therapeutically changeable state. This view supports integrating epigenetic and metabolic interventions into microglial state-calibration (31, 49).

### 2.3 LDAM, immunosenescence, and SASP-like microglial dysfunction

Lipid droplet-accumulating microglia should not be interpreted merely as cells with abnormal lipid storage. LDAM-like states may partially overlap with broader concepts of microglial immunosenescence and senescence-associated secretory phenotype (29, 69). In aged and *APOE* ϵ4 Alzheimer’s brains, exhausted-like microglia exhibit lipid accumulation, reduced phagocytic competence, increased oxidative stress, and inhibitory receptor expression, such as TIM-3, as well as inflammatory secretory programs (29). These features resemble a senescence-like state in which cells are metabolically overloaded, poorly adaptive, and chronically inflammatory (29, 69).

This view strengthens the rationale for therapies that target aging biology, lysosomal function, and autophagy (70–72). Rapamycin, metformin, NAD+ restoration strategies, mitochondrial protectants, and lysosome-supporting interventions may not simply reduce inflammation; they may counteract microglial immunosenescence (41, 70–74). However, the timing of the intervention is crucial. In late LDAM-like states, lysosomes may already be overloaded, and indiscriminate activation of autophagy could worsen intracellular stress unless lysosomal clearance capacity is restored first (70–72).

### 2.4 Biological sex as a modifier of microglial state transitions

Biological sex is a crucial and often under-integrated determinant of microglial state dynamics in AD (29–31). Sex differences in microglia originate from the combined effects of sex chromosomes, gonadal hormones, aging-related endocrine changes, and genotype-specific risk modifiers (29, 30). Female and male microglia consistently differ in basal immune tone, phagocytic thresholds, interferon responsiveness, complement activity, lipid-handling capacity, and vulnerability to senescence-like remodeling (29–31). These differences directly impact AD pathology.

Sex-specific differences in microglial dynamics directly influence AD risk, biomarker progression, and response to therapy. These distinctions indicate that sex is a critical biological modifier, affecting microglial phenotype assignment, biomarker interpretation, and therapeutic calibration in AD trials. Integrating sex as a core variable rather than a demographic covariate is essential to advancing precision in AD research (31).

## 3 Microglial heterogeneity and spatiotemporal state development at the single-cell omics level

Microglia in AD are not a single type. Recent advances—including single-cell and single-nucleus RNA sequencing, spatial transcriptomics, and humanized microglial models—have shown varied activation states, regions, and stage changes (22–28, 75, 76). These technologies also reveal major interspecies differences, which make translation from mouse models to human AD harder (23, 25–28, 76, 77).

### 3.1 Disease-associated microglia programs and microenvironment-dependent activation

The discovery of disease-associated microglia was a milestone in Alzheimer’s immunology (58). In plaque-bearing mouse models, some microglia reduce P2ry12 and Tmem119. When TREM2 is active, these cells increase Apoe, Lpl, Clec7a, Cst7, and other genes involved in lipid handling and debris clearance (58, 59, 78). This shift is spatial: microglia near plaques show greater plaque-related activity, while distant cells may lack typical DAM markers but can still exhibit age-related changes, blood vessel dysfunction, or myelin abnormalities (25, 29, 69, 78–80).

DAM should not be seen as always protective or always harmful. Early DAM-like activation may help compact plaques and clear Aβ. Chronic or late-stage activation can lead to neurodegenerative, inflammatory, lipid-filled, or tired states (29, 58, 59, 69, 78–80). Disease stage, APOE genotype, tau burden, vascular problems, and local tissue injury all affect whether a DAM-like response is helpful or harmful. (**Table 2**) summarizes key microglial states, their markers, disease-stage associations, and locations.

**Table 2:** High-dimensional microglial states in Alzheimer’s disease and their spatiotemporal features.

High-dimensional microglial states in Alzheimer’s disease and their spatiotemporal features				
Microglial state	Representative transcriptional/surface markers	Disease stage and spatial niche	Core functional phenotype	Reference(s)
Homeostatic microglia	*P2RY12+*, *TMEM119+*, *CX3CR1+*, *HEXB+*	Healthy brain and very early Alzheimer’s disease; broadly distributed in the parenchyma and typically distant from plaques.	Physiologic immune surveillance, synaptic remodeling through the *CX3CR1* axis, and trophic support for tissue homeostasis.	(22, 47, 57)
Disease-associated microglia (DAM; stage 1/2)	TREM2 high, *APOE* high, *LPL+*, *CLEC7A+*, *CST7+*	Early Alzheimer’s disease; concentrated around amyloid-beta plaques and exhibiting a plaque-proximal niche effect (<50 μm).	TREM2-dependent activation program with enhanced lipid handling, plaque compaction, and amyloid-beta phagocytosis; generally protective in the early phase.	(58, 59, 78–80)
Microglia in neurodegeneration (MGnD)	*APOE*+++, *BHLHE40*+, *CLEC7A*+, *SPP1*+, *CD74*+	Middle-to-late disease stages; enriched near tau pathology and regions with pronounced neuronal degeneration.	Pro-inflammatory and neurotoxic phenotype with loss of beneficial phagocytic support; promotes complement-driven synaptic stripping through the C1q/C3b axis.	(32–36, 59, 81–85)
Interferon-responsive microglia (IRM)	*IFIT1*+, *IFITM3+*, *STAT1+*, *CXCL10*+, *ISG15*+	Observed across disease stages in cortex and hippocampus, likely driven by nucleic acid leakage and interferon-rich microenvironments.	Type I interferon-like response program that amplifies and sustains chronic neuroinflammation.	(23, 27, 86–91)
Lipid droplet-accumulating/exhausted microglia (LDAM)	BODIPY lipid staining+, TIM-3+, *ACSL1*+, *PLIN2*+	Late-stage Alzheimer’s disease and aging brain; especially enriched in *APOE* ϵ4 homozygous carriers.	Severe phagocytic paralysis, lipid metabolic deadlock, increased reactive oxygen species generation, and senescence-associated secretory activity.	(29, 32, 69, 70, 74)

Microglial states are presented as a dynamic continuum rather than as mutually exclusive categories. Marker sets are representative rather than exhaustive.

ACSL1, acyl-CoA synthetase long chain family member 1; AD, Alzheimer’s disease; APOE, apolipoprotein E; BHLHE40, basic helix-loop-helix family member e40; BODIPY, boron-dipyrromethene (fluorescent dye); C1q, complement component 1q; C3b, complement component 3b; CD74, CD74 molecule; CLEC7A, C-type lectin domain containing 7A; CR3, complement receptor 3; CST7, cystatin F; CX3CR1, C-X3-C motif chemokine receptor 1; CXCL10, C-X-C motif chemokine ligand 10; DAM, disease-associated microglia; HEXB, hexosaminidase subunit beta; IFIT1, interferon induced protein with tetratricopeptide repeats 1; IFITM3, interferon induced transmembrane protein 3; IRM, interferon-responsive microglia; ISG15, ISG15 ubiquitin-like modifier; LDAM, lipid droplet-accumulating microglia; LPL, lipoprotein lipase; MGnD, microglia in neurodegeneration; P2RY12, purinergic receptor P2Y12; PLIN2, perilipin 2; SPP1, secreted phosphoprotein 1; STAT1, signal transducer and activator of transcription 1; TIM-3, T-cell immunoglobulin and mucin domain containing 3; TMEM119, transmembrane protein 119; TREM2, triggering receptor expressed on myeloid cells 2.

#### 3.1.1 Distal microglia are not necessarily healthy bystanders

Although plaque-proximal microglia have received the most attention, distal microglia are biologically significant. Cells located far from amyloid plaques may appear relatively quiescent by plaque-associated markers, yet they are not healthy bystanders (22, 29, 69, 80). In the aged brain, distal microglia undergo Aβ-independent intrinsic aging, reduced surveillance motility, altered lipid handling, impaired mitochondrial function, and diminished support for white-matter integrity (22, 69, 74).

This broader interpretation suggests that diffuse gliosis in AD involves microglial changes triggered not only by Aβ plaques but also by age-driven tissue vulnerability, vascular injury, myelin stress, and systemic metabolic dysfunction (22, 29, 69, 74). As a result, targeting only plaque-proximal microglia is insufficient; distal microglial aging programs also play a critical role in network-level cognitive decline, emphasizing the need for more comprehensive therapeutic strategies.

### 3.2 Challenges in clinical translation: species-specific gaps

Transgenic mouse models are vital for studying disease mechanisms, but their microglial states do not match those found in sporadic human AD (23, 76, 77). Mouse atlases, primarily based on strong familial mutation models, do not reflect sporadic disease, which develops over years and is shaped by age, vascular damage, other health conditions, APOE genotype, sex, and environmental factors (29–31, 77).

Human snRNA-seq and xenografted human microglial models show that human Alzheimer’s microglia differ substantially from mouse DAM (25–28, 76). Human cells often display stronger relationships with tau pathology, lipid dysregulation, inflammatory signaling, and disease-risk genes such as PLCG2, ABCA7, INPP5D, and APOE (14, 19, 20, 27, 28, 86, 92, 93). Mouse DAM markers, such as Clec7a and Lpl, may indicate plaque proximity and activation in mice, but they should not be automatically interpreted as causal drivers of human disease (23, 25–28). In the human brain, some DAM-like markers may function as context-dependent correlates of local pathology rather than conserved therapeutic targets (25–28).

Microglia-targeting therapies must be validated in human iPSC-derived microglia, chimeric mouse models with human microglia, organoid or assembloid systems, post-mortem spatial omics, and patient-derived biomarker datasets (27, 75, 76). **Supplementary Table 1** explicitly outlines a structured certainty assessment for each mechanistic claim, distinguishing evidence from mouse models, human post-mortem tissue, iPSC-derived or humanized microglial systems, and clinical biomarker or trial data.

## 4 Core hubs driving microglial state changes: receptor and metabolic networks

**Figure 1:**
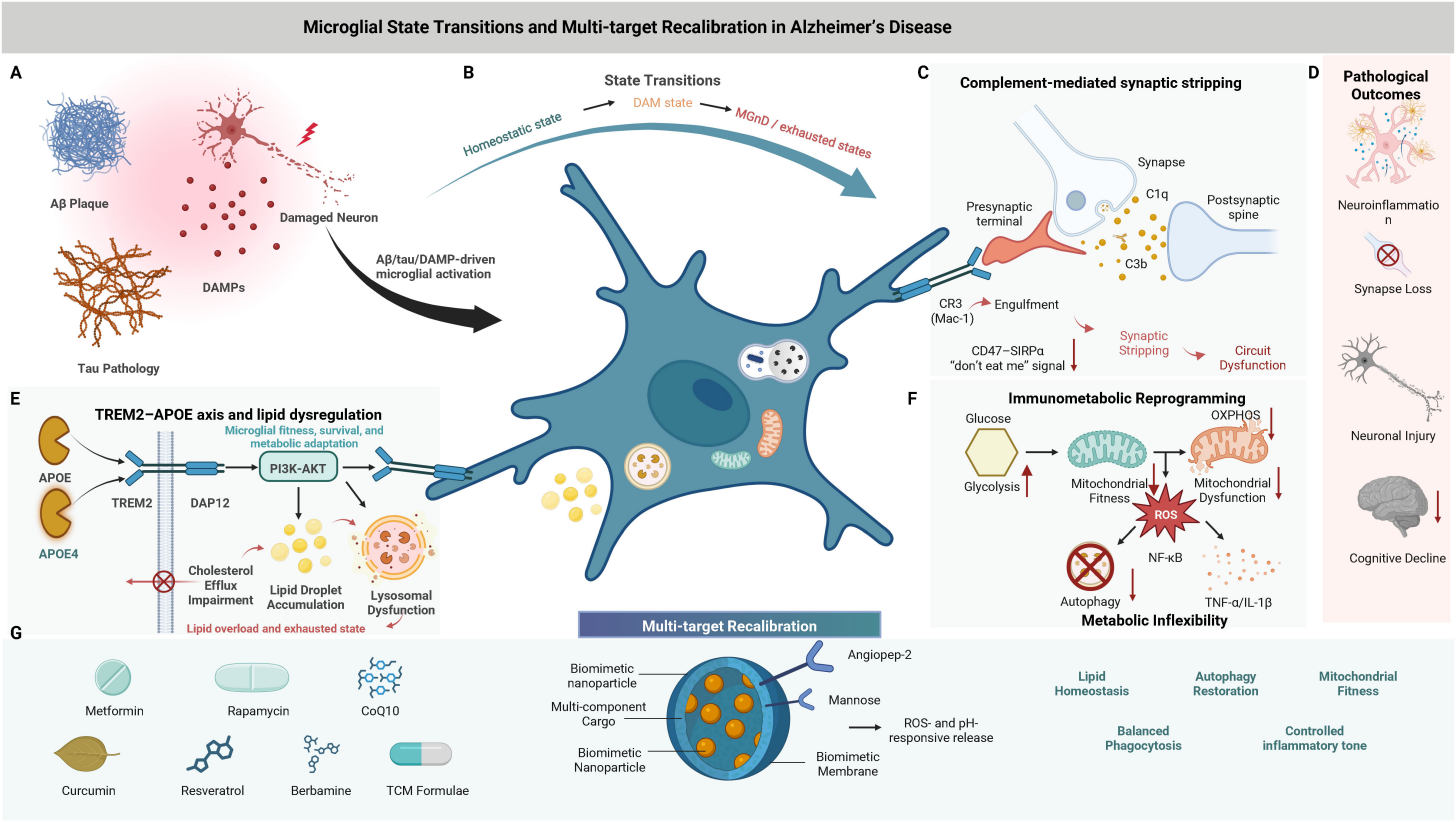
Microglial state transitions and multi-target recalibration in Alzheimer’s disease. **(A)** Amyloid-β (Aβ) plaques, tau pathology, and damage-associated molecular patterns (DAMPs) released from injured neurons initiate microglial activation in the Alzheimer’s disease (AD) microenvironment. **(B)** Activated microglia undergo progressive state transitions from a homeostatic state to disease-associated microglia (DAM), and with persistent pathological stress may further shift toward microglia in neurodegeneration (MGnD) or exhausted states. **(C)** Complement-mediated synaptic stripping is driven by deposition of C1q and C3b on vulnerable synapses, followed by recognition through complement receptor 3 (CR3; Mac-1) on microglia. Loss of the CD47–signal regulatory protein alpha (SIRPα) “don’t eat me” signal further facilitates aberrant engulfment, leading to synaptic stripping and circuit dysfunction. **(D)** These state transitions collectively contribute to major pathological outcomes, including neuroinflammation, synapse loss, neuronal injury, and cognitive decline. **(E)** The triggering receptor expressed on myeloid cells 2 (TREM2)–apolipoprotein E (APOE) axis is a central regulator of lipid sensing and metabolic adaptation. Through DNAX-activating protein of 12 kDa (DAP12) and phosphoinositide 3-kinase/protein kinase B (PI3K–AKT) signaling, TREM2 supports microglial fitness, survival, and metabolic adaptation. In the *APOE*4 context, impaired cholesterol efflux promotes lipid droplet accumulation and lysosomal dysfunction, driving lipid overload and exhausted microglial states. **(F)** Immunometabolic reprogramming in AD is characterized by increased glycolysis, reduced oxidative phosphorylation (OXPHOS), mitochondrial dysfunction, reactive oxygen species (ROS) accumulation, impaired autophagy, and activation of nuclear factor kappa B (NF-κB)-associated inflammatory signaling, resulting in metabolic inflexibility and increased production of tumor necrosis factor alpha (TNF-α) and interleukin-1 beta (IL-1β). **(G)** The lower panel summarizes a proposed multi-target recalibration strategy integrating metabolic modulators, natural bioactive compounds, traditional Chinese medicine (TCM) formulae, and biomimetic nanoparticle-based delivery systems. Surface modification with angiopep-2 and mannose, together with ROS- and pH-responsive release, is designed to improve brain delivery and microglial targeting. These interventions aim to restore lipid homeostasis, balanced phagocytosis, autophagy, mitochondrial fitness, and controlled inflammatory tone. Aβ, amyloid-beta; AD, Alzheimer’s disease; AKT, protein kinase B; APOE, apolipoprotein E; *APOE*4, apolipoprotein E epsilon 4; C1q, complement component 1q; C3b, complement component 3b; CR3, complement receptor 3; DAM, disease-associated microglia; DAP12, DNAX-activating protein of 12 kDa; DAMPs, damage-associated molecular patterns; IL-1β, interleukin-1 beta; Mac-1, macrophage-1 antigen; MGnD, microglia in neurodegeneration; NF-κB, nuclear factor kappa B; OXPHOS, oxidative phosphorylation; pH, potential of hydrogen; PI3K, phosphoinositide 3-kinase; ROS, reactive oxygen species; SIRPα, signal regulatory protein alpha;TCM, traditional Chinese medicine; TNF-α, tumor necrosis factor alpha; *TREM2*, triggering receptor expressed on myeloid cells 2; CoQ10, coenzyme Q10.

**Figure 1** illustrates the integrated nature of these pathological modules. Specifically, it depicts the TREM2–APOE lipid-sensing axis, complement-mediated synaptic stripping, immunometabolic reprogramming, and a proposed multi-target recalibration strategy. Building on these modules, the following discussion focuses on the interconnected signaling and metabolic hubs driving microglial state changes in AD.

In AD, microglial transitions toward neurodegenerative or exhausted states are driven by interconnected signaling and metabolic hubs. The literature consistently identifies three major axes: TREM2–APOE lipid sensing (32, 58, 59), complement-mediated synaptic clearance (33–36), and immunometabolic reprogramming (37–41). To better understand their interplay, the next paragraphs examine how these axes interact within microglial networks.

These axes connect. Lipid overload disrupts receptor signaling and phagocytosis (29, 32, 69). Complement activation changes pruning thresholds. Mitochondrial stress and glycolysis affect cytokine output (37–41, 74). Chronic inflammation harms lipid and lysosomal balance (11, 61, 70). Therapies should target microglial state networks rather than single nodes (12, 13, 31, 42–44).

### 4.1 The TREM2–APOE signaling axis and lipid metabolic imbalance

TREM2 is a decisive damage- and lipid-sensing receptor in microglia (15, 18, 32). In plaque-associated microglia, TREM2 sustains survival, lipid processing, lysosomal function, and Aβ phagocytosis (32, 58, 78, 94). When triggered, TREM2 signals via DAP12, activating PI3K–AKT to secure microglial adaptation under amyloid stress (32, 94). Loss-of-function TREM2 mutations sharply elevate AD risk (15, 18), while TREM2 disruption halts microglial adaptation toward plaque-associated survival states (78, 94).

Building on these functions, APOE drives this axis (32, 59). Normally, astrocytes produce brain APOE, but in AD, activated microglia boost APOE transcription (59). APOE ϵ4 disrupts cholesterol efflux and lipid homeostasis, leading to lipid droplet accumulation, reduced phagocytosis, inflammatory dysfunction, and progression to LDAM-like exhaustion (29, 32, 69, 87, 95).

Taken together, these findings suggest that restoring microglial lipid homeostasis may be more rational than simply increasing microglial activation (29, 32, 87, 95). The aim should be to preserve lipid handling, lysosomal clearance, and controlled phagocytosis without pushing microglia toward chronic inflammatory activation (31, 32, 70).

#### 4.1.1 Lipid overload as a driver of lysosomal congestion and phagocytic failure

A key unresolved issue is how a lipid-sensing program transitions from adaptive to maladaptive during disease progression. In early plaque-associated microglia, TREM2-dependent lipid uptake may support membrane remodeling, plaque compaction, and phagocytosis (32, 58, 78). However, when lipid influx exceeds the capacity of cholesterol efflux, lysosomal degradation, and mitochondrial oxidation, these processes become congested (29, 69, 70, 95). This imbalance can lead to lipid droplet accumulation, endolysosomal swelling, impaired lysosomal acidification, and reduced degradation of engulfed Aβ or myelin-derived debris (69, 70).

This transition is particularly relevant in APOE ϵ4 carriers. APOE ϵ4-associated defects in astrocyte–microglia lipid transport and cholesterol efflux may reduce the ability of microglia to export cholesterol and resolve lipid stress (29, 32, 87, 95). As a result, microglia may remain locked in a state of partial activation: they continue to sense plaques and inflammatory danger signals but lose efficient clearance capacity (29, 69, 70). This state may be characterized by reduced phagocytosis, elevated oxidative stress, and persistent release of inflammatory mediators (29, 69, 70). Therefore, lipid overload should not be viewed merely as a metabolic by-product; it may actively drive microglial state drift from protective plaque containment toward lysosomal exhaustion and inflammatory persistence (29, 69, 70, 95).

Therapeutically, this suggests that restoring lipid homeostasis may require concurrent receptor modulation. TREM2 activation may be beneficial only when lipid processing, lysosomal degradation, and cholesterol efflux pathways are functional (31, 32, 94). If microglia are lipid-overloaded or lysosome-compromised, stimulating TREM2 may increase substrate uptake without improving degradation. This can aggravate intracellular stress (29, 31, 70). This provides a mechanistic explanation for why receptor agonism alone may be insufficient in clinically heterogeneous AD populations (31, 96–98).

#### 4.1.2 The Goldilocks principle of TREM2 signaling

TREM2 signaling follows a Goldilocks-like principle: too little signaling harms, while excessive or mis-timed signaling is also maladaptive (15, 18, 31, 32). Loss-of-function variants such as R47H reduce ligand binding, impair plaque-associated microglial responses, and heighten AD risk (15, 18). However, unconditional TREM2 stimulation does not automatically yield clinical benefit. The phase II failure of the TREM2 agonistic antibody AL002 (97–100), despite evidence of target engagement and pharmacodynamic activity in earlier studies, demonstrates that receptor activation alone cannot guarantee disease modification (98).

One possible explanation is that the therapeutic effect of TREM2 depends on microglia’s downstream capacity to process lipid cargo, maintain lysosomal flux, resolve inflammation, and avoid collateral synaptic pruning (29, 31, 32, 70). In lipid-overloaded or lysosome-compromised microglia, further receptor stimulation may increase substrate uptake without improving degradation, thereby aggravating intracellular stress. The therapeutic goal should therefore not be “more TREM2 signaling,” but appropriately timed and state-dependent TREM2 signaling within an optimal window defined by disease stage, APOE genotype, inflammatory tone, plaque burden, and microglial metabolic capacity (31, 96, 98).

### 4.2 Complement overactivation cascades and pathological synaptic pruning

Synaptic loss is an early, clinically relevant feature of AD, affecting the hippocampus and entorhinal cortex (33, 82). Microglia reactivate complement-dependent synaptic pruning pathways during this process (33–36). In development, C1q/C3-mediated and CR3-dependent phagocytosis refine neural circuits (35, 36). In AD, oligomeric Aβ and inflammatory cues trigger excessive deposition of microglia-derived C1q on vulnerable synapses (33, 34, 55). This activates the classical complement pathway, leading to C3 cleavage and deposition of C3b/iC3b. Opsonized synapses are then recognized by microglial CR3/Mac-1 and removed (33–36).

This mechanism shows synaptic loss as an active neuron-glia immune process, not a passive result of neuronal death (33, 82). Interrupting key complement components reduces synapse loss in experimental models. Complement-mediated pruning is an attractive therapeutic target (33, 34).

#### 4.2.1 Protective molecular brakes against excessive pruning

Complement negative regulators, such as CD55 and CD59, limit complement amplification and the formation of the membrane attack complex (34). If reduced or overwhelmed in AD, complement activation may become excessive and synaptotoxic (33, 34).

Building on this, the CD47–SIRPα “don’t eat me” pathway provides an anti-phagocytic signal that protects viable cells and synapses from inappropriate engulfment (83, 84). Downregulation or functional impairment of this axis can remove inhibitory constraints on microglial phagocytosis and promote synaptic stripping (83, 84).

These brakes collectively define a set-point for synaptic preservation, but the fate of a synapse depends on the balance between protective and opposing “eat me” signals. Thus, complement therapy should not indiscriminately suppress complement activity. Complete complement blockade risks impairing debris clearance, host defense, and synaptic remodeling (34–36). Therapy, instead, aims to restore pruning thresholds so that microglia clear damaged synapses and debris while preserving functional synapses (33, 34, 82). Modulation of the PI3K/AKT/GSK3β axis has been proposed as a targeted approach to reinforce neuroprotective signals and limit excessive complement-driven synaptic elimination (85).

#### 4.2.2 The unresolved challenge of selective phagocytosis

A central therapeutic challenge is that microglial phagocytosis is not automatically restricted to pathological substrates (63, 64, 82). The same cellular machinery that removes Aβ aggregates, apoptotic cells, and dystrophic neurites can also eliminate stressed but viable synapses if inhibitory signals are weakened and opsonization thresholds are lowered (33, 82–84). Therefore, increasing microglial phagocytosis is not automatically beneficial; the therapeutic goal should be to improve phagocytic precision (63, 64, 82).

As introduced in 4.2.1, synaptic fate depends on the local balance between “eat me” and “don’t eat me” signals (83, 84). “Eat me” signals arise from pathological substrates—aggregated Aβ, damaged membranes, and degenerating neurites—that display key features: abnormal lipid composition, complement deposition, phosphatidylserine exposure, and antibody-bound immune complexes, which promote engulfment (33, 34, 82). Conversely, “don’t eat me” protective signals, such as CD47–SIRPα and complement regulators, help functional synapses resist engulfment (83, 84). A successful therapy would boost recognition of pathological signals while preserving protective mechanisms to maintain a high threshold for engulfment of viable synaptic structures (33, 82–84).

This issue is especially relevant to antibody-based amyloid clearance. Anti-Aβ antibodies promote Fc receptor-mediated uptake of amyloid-containing material. They may also modify local complement activation and microglial phagocytic thresholds (8–10, 33, 34). In an inflammatory, APOE ϵ4-associated, or complement-primed microenvironment, antibody-induced clearance could theoretically increase collateral synaptic vulnerability (29, 32–34). Synapse-protective strategies should be considered companions to amyloid clearance, not alternatives (12, 13, 82).

### 4.3 Immunometabolic reprogramming, mitochondrial dysfunction, and metabolic rigidity

Microglial inflammation is closely tied to cell metabolism (37–40). In early AD, increased glycolysis may help phagocytosis, migration, cytokine release, and stress adaptation (37, 38). As AD advances, ongoing Aβ exposure, tau injury, lipid excess, and chronic inflammation may fix microglia in a rigid metabolic state, marked by aerobic glycolysis, fragmented mitochondria, increased reactive oxygen species, impaired mitophagy, dysfunctional lysosomes, depleted NAD+, and inflammatory fatigue (37–41, 69, 70, 74).

#### 4.3.1 HIF-1α/PKM2 signaling and Warburg-like glycolytic reprogramming

A key molecular switch sustaining glycolytic reprogramming is stabilization of hypoxia-inducible factor-1α. HIF-1α promotes transcription of glycolytic genes, including glucose transporters and glycolytic enzymes, and supports inflammatory cytokine production. In microglia exposed to chronic inflammatory or amyloid stress, HIF-1α stabilization can create a brain-resident analog of the Warburg effect: cells preferentially rely on glycolysis even when oxygen is available (37, 40, 101).

Pyruvate kinase M2 is another important regulator. PKM2 controls the final rate-limiting step of glycolysis and can also translocate to the nucleus, where it cooperates with HIF-1α and inflammatory transcriptional programs (102). The HIF-1α/PKM2 module may therefore serve as a feed-forward circuit: inflammation stabilizes HIF-1α, HIF-1α enhances glycolysis, glycolysis supports the production of inflammatory mediators, and PKM2 reinforces this transcriptional-metabolic loop (101, 102). This creates a central Warburg-like state in which microglia become efficient inflammatory producers but progressively lose oxidative flexibility and homeostatic resilience (37–40).

#### 4.3.2 AMPK–mTORC1–ULK1 crosstalk and autophagy failure

The AMPK–mTORC1–ULK1 axis constitutes a pivotal energy-sensing pathway integrating nutrient status, mitochondrial function, and autophagy (103, 104). Under conditions of energy stress, AMPK activation facilitates catabolic restoration. AMPK directly phosphorylates ULK1, notably at Ser555, thereby initiating autophagy (103, 104). Concurrently, AMPK suppresses mTORC1, a principal negative regulator of autophagy, by phosphorylating TSC2 and Raptor (103–105). This dual mechanism not only activates ULK1 but also alleviates mTORC1-mediated inhibition of autophagy (103–105). Thus, the coordinated activity of these molecules tightly regulates cellular energy responses.

Disruption of this regulation is evident in AD. Persistent metabolic stress, insulin resistance-like signaling, mitochondrial injury, and inflammatory activation can impair adaptive AMPK signaling or produce maladaptive mTORC1 activity (40, 41, 106). As a result, autophagy initiation, autophagosome maturation, lysosomal acidification, and substrate degradation become uncoupled (70, 72, 106). Recent work emphasizes that disruption of this metabolic-autophagic balance contributes to Alzheimer’s-associated neuronal and glial dysfunction (41, 106). This supports therapeutic strategies using metformin, rapamycin, or related metabolic modulators, but also highlights the need for careful timing and biomarker-guided dosing (41, 106–108).

#### 4.3.3 Why metabolic switching becomes irreversible: mtDNA damage and NAD+ depletion

A major unresolved question is why adaptive glycolytic switching becomes chronic and hard to reverse; two types of molecular damage may cause this persistence.

Mitochondrial reactive oxygen species can damage mitochondrial DNA, which encodes key subunits of the electron transport chain. When persistent oxidative damage or mutations impair oxidative phosphorylation (74, 109), respiratory capacity declines, forcing microglia to maintain glycolytic compensation even if inflammation subsides (37–40, 74).

Second, chronic oxidative DNA damage activates DNA repair enzymes such as PARP-1. Excessive PARP-1 activation consumes intracellular NAD+, a critical cofactor for redox metabolism, mitochondrial function, sirtuin activity, and DNA repair (110, 111). Chronic NAD+ depletion weakens mitochondrial oxidative metabolism and reduces cells’ capacity to restore metabolic flexibility (110, 111). Thus, mtDNA injury and NAD+ exhaustion may convert a reversible metabolic adaptation into a self-perpetuating metabolic deadlock (74, 110, 111).

#### 4.3.4 mtDNA leakage, cGAS–STING activation, and interferon-responsive microglia

Mitochondrial damage links metabolism to innate immune signaling. If damaged mitochondria are not cleared or if their membranes are permeabilized, mtDNA accumulates in the cytosol (88, 90). cGAS senses cytosolic mtDNA, produces cyclic GMP-AMP, and activates STING (88, 91). STING then induces type I interferon responses and inflammatory transcriptional programs (89, 91).

This mechanism provides a plausible bridge between mitochondrial dysfunction and interferon-responsive microglial states described in single-cell studies (23, 27, 86, 87). IRM-like cells expressing IFIT1, IFITM3, STAT1, CXCL10, and ISG15 may therefore arise, at least in part, from mitochondrial damage and mtDNA-driven innate immune activation (23, 27, 86–88, 91). In this model, impaired mitochondrial quality control not only reduces energy production but can also transform damaged mitochondria into endogenous inflammatory triggers (88–91).

#### 4.3.5 Therapeutic implications

Immunometabolic therapy should focus on restoring metabolic flexibility instead of solely suppressing inflammation. Strategies include AMPK activation, mTORC1 normalization, lysosome restoration, NAD+ replenishment, mitochondrial antioxidants, enhanced mitophagy, and inhibition of maladaptive signaling pathways (41, 70–72, 88–91, 101–106, 110–116). Interventions must be tailored to the disease phase: early glycolytic activity may support protective phagocytosis, whereas persistent glycolytic rigidity later can drive inflammatory exhaustion (37–40, 69, 74). Metabolic therapies require calibration through suitable biomarkers (31, 73, 117).

#### 4.3.6 Stage-dependent windows for immunometabolic intervention

The same metabolic intervention can have varying consequences depending on disease stage and microglial state. In early plaque-associated microglia, moderate glycolytic activation may promote migration, phagocytosis, and plaque containment (37–40, 58, 78–80), so indiscriminate suppression of glycolysis at this stage could weaken protective clearance responses. By contrast, in late-stage, lipid-overloaded or senescent-like microglia, persistent glycolysis tends to indicate metabolic rigidity rather than adaptation (37–40, 69, 74, 106, 117). In this context, restoring mitochondrial oxidative capacity, NAD+ availability, lysosomal competence, and mitophagic flux is likely more beneficial than further stimulating inflammatory metabolism (41, 70–74, 103–111, 113, 114).

This stage dependency has direct therapeutic implications. While AMPK activation may support autophagy and mitochondrial quality control, excessive activation in energy-depleted cells could increase catabolic stress (41, 103–108). Although mTORC1 inhibition may promote autophagy initiation, if lysosomal degradation is impaired, autophagosome accumulation may worsen cellular dysfunction (70–72, 103–106, 112, 115, 116). NAD+ replenishment may improve redox balance and sirtuin activity, but its efficacy may depend on whether mitochondrial networks can recover structurally (110, 111). Similarly, cGAS–STING inhibition may reduce interferon-driven inflammation, yet broad suppression of innate immune sensing may impair host defense and debris clearance (88–91).

Immunometabolic therapy should be guided by biomarkers rather than applied uniformly. Potential biomarker combinations include CSF or plasma inflammatory cytokines, sTREM2, GFAP, NfL, metabolic markers related to lactate or NAD+ status, lipidomic signatures, mitochondrial injury markers, and imaging readouts of neuroinflammation (31, 73, 117, 118). The central goal is not to force microglia into a predetermined “anti-inflammatory” phenotype. Instead, restore metabolic flexibility and the ability to switch between surveillance, clearance, repair, and resolution (37–40, 64, 65, 117).

#### 4.3.7 Integrated view: the immunometabolic vicious cycle in AD microglia

The metabolic nodes described above are self-reinforcing: chronic Aβ and tau stress stabilize HIF-1α, which directly promotes PKM2-driven glycolysis (37, 40, 101, 102). This metabolic shift suppresses AMPK, permitting aberrant mTORC1 activity, which uncouples the AMPK–mTORC1–ULK1 axis and thereby disrupts autophagic flux (41, 103–106). As autophagic flux is impaired, mitophagy falters, causing the accumulation of damaged, ROS-generating mitochondria. Once these mitochondria permeabilize, oxidized mtDNA leaks into the cytosol, activating cGAS–STING signaling (88–91). This activation triggers a type I interferon response, which amplifies neuroinflammation and further inhibits autophagic clearance, closing a positive feedback loop (88, 91). Chronic mitochondrial ROS directly injures mtDNA, and persistent DNA damage activates PARP-1. These events deplete NAD+, reducing both oxidative metabolism and sirtuin-mediated repair (110, 111). Altogether, these processes trap microglia in a state defined by high glycolysis, low respiration, and persistent inflammation (37–40, 74). This model clarifies why single-node interventions are insufficient: each intervention disrupts only a single component of a network reinforced by multiple, mutually locking mechanisms.

## 5 Network pharmacology and multi-target synergistic interventions

For microglia, the main intervention is to prevent harmful state drift and maintain their capacity for immune surveillance, phagocytosis, synaptic support, and tissue repair (31, 42, 64, 65, 117).

### 5.1 Fine-tuning core signaling pathways and restoring metabolic function

Therapies targeting TREM2 illustrate both the promise and limitations of receptor-focused strategies. AL002 showed target engagement and increased soluble TREM2 in early studies (97, 100), but the phase II trial did not meet its primary endpoint (98). This suggests that activating one receptor cannot overcome the broader disease network if lipid overload, complement dysregulation, mitochondrial dysfunction, and cellular exhaustion are already advanced.

Metabolic interventions may provide a more systemic approach. Metformin activates AMPK, impacting autophagy, inflammation, and insulin-related signaling (41, 107, 108, 119, 120). Rapamycin inhibits mTORC1 and promotes autophagy, with effects influenced by lysosomal competence and dosing schedule (70–72, 103–106, 115, 116). Coenzyme Q10 supports mitochondrial redox balance (113, 114). Resveratrol modulates SIRT1-linked pathways, inflammation, mitochondrial quality control, and may affect proteostasis (121–123). (**Table 3**) highlights clinical programs directly relevant to microglial state-calibration. **Supplementary Table 2** provides a broader list of clinical development programs targeting microglial signaling, neuroinflammation, immunometabolism, and related pathways (152–169).

**Table 3:** Selected clinical programs informing microglial state-calibration in Alzheimer’s disease.

Selected clinical programs informing microglial state-calibration in alzheimer’s disease						
Category	Target/pathway	Agent	Trial/clinical status	Key findings/remarks	Link to the review’s mechanistic axes	Reference(s)
Receptor modulation	TREM2	AL002	Phase I completed; Phase II completed/unsuccessful; extension terminated	Demonstrated target engagement and increased CSF sTREM2, but phase II did not meet primary endpoint; ARIA among common adverse events	Directly linked to TREM2–APOE lipid-sensing axis; supports calibration rather than unconditional agonism	(97–100)
Receptor modulation	TREM2	VG-3927	Phase I	Designed to assess safety and target engagement, including CSF sTREM2	TREM2 signaling window and lipid/phagocytic adaptation	(124)
Metabolic modulation	AMPK	Metformin	Phase II completed; prevention trial ongoing	Reported cognitive or biomarker signals in selected studies; activates AMPK and may modulate NF-κB and autophagy	Immunometabolic axis; AMPK–mTOR–ULK1 recalibration	(41, 107, 108, 119, 120)
Autophagy/kinase	mTOR	Rapamycin	Phase I completed; Phase II ongoing	Tests feasibility of mTOR inhibition and autophagy modulation; requires caution regarding autophagy stress	Immunometabolic/autophagy axis; LDAM and immunosenescence	(103–106, 112, 115, 116)
Natural product	SIRT1/metabolic-inflammatory pathways	Resveratrol	Phase II completed	Reduced CSF MMP9 and Aβ40 in reported trial; broad pleiotropic effects but bioavailability remains limiting	Immunometabolic and inflammatory regulation; network-level modulation	(121–123)
Growth factor/immunomodulation	GM-CSF	Sargramostim	Phase II completed/ongoing	Reported cognitive and biomarker changes in early studies; further validation needed	Immune recalibration and phagocytic support	(125–127)
Tau-directed adjunct	MAPT/tau	NIO752	Phase I ongoing	Intrathecal antisense strategy to reduce tau synthesis	Not a microglial target, but relevant to downstream tau-driven microglial activation and MGnD transition	(128)

This table highlights representative programs most directly relevant to the mechanistic axes discussed in this review. A more comprehensive list is provided in **Supplementary Table 2**. AD, Alzheimer’s disease; aMCI, amnestic mild cognitive impairment; AMPK, AMP-activated protein kinase; APOE, apolipoprotein E; ARIA, amyloid-related imaging abnormalities; CSF, cerebrospinal fluid; GM-CSF, granulocyte-macrophage colony-stimulating factor; MAPT, microtubule-associated protein tau; mTOR, mechanistic target of rapamycin; NF-κB, nuclear factor kappa B; SIRT1, sirtuin 1; sTREM2, soluble triggering receptor expressed on myeloid cells 2; *TREM2*, triggering receptor expressed on myeloid cells 2.

#### 5.1.1 Additional network hubs beyond TREM2

A robust multi-target framework must extend beyond TREM2. Multiple inflammatory and resolution-related hubs require focused attention.

First, consider the inhibitory immune checkpoint pathways, such as TREM2/LILRB4-related modules, which may regulate myeloid restraint, phagocytosis, and chronic inflammatory adaptation. Although such axes are better characterized in peripheral immune settings, they may provide useful analogies for restraining excessive microglial activation (64, 65, 117).

Next, the NLRP3 inflammasome acts as a metabolic-inflammatory sensor. It integrates mitochondrial ROS, lysosomal rupture, ionic stress, Aβ-related danger signals, and inflammatory priming (129). NLRP3 inhibition may therefore reduce IL-1β production while also interrupting metabolic-injury feedback loops (129, 130).

Finally, pro-resolving GPCRs, including CB2 and FPR2, may shift microglia from inflammatory amplification toward resolution, efferocytosis, and tissue repair. These pathways are attractive because they may not simply block inflammation but actively promote resolution. Recent pharmacological reviews support a systemic multi-target approach integrating inflammatory checkpoints, metabolic sensors, and GPCR-mediated resolution pathways (42, 43, 131).

#### 5.1.2 Autophagy stress: a necessary warning

Autophagy restoration offers potential benefits but carries clear risks. In LDAM-like or senescent microglia, lysosomal function is often compromised. Strongly inducing autophagy when lysosomes cannot degrade cargo will cause autophagosome accumulation, escalate cellular stress, and trigger apoptosis or inflammatory cell death (70–72, 112, 117).

The treatment sequence must be optimized. In late-stage lipid-overloaded microglia, first restore lysosomal acidification, cholesterol efflux, and degradative capacity before inducing autophagy (70–72, 95, 112, 117). Apply this approach especially with rapamycin-like therapies or natural products intended to “activate autophagy” (112, 115, 116, 132). Ensure restoration of autophagic flux, not just increased autophagosome formation.

### 5.2 Multicomponent natural therapies and smart nanodelivery

Natural bioactive molecules such as curcumin, berberine, and resveratrol are attractive because they modulate multiple pathological processes, including inflammation, oxidative stress, mitochondrial dysfunction, impaired autophagy, and lipid imbalance (133–135). Traditional Chinese medicine formulas such as Liuwei Dihuang Pill and Yishen Huazhuo decoction have shown preclinical effects in AD models, including reduced glial activation, altered TREM2/NF-κB signaling, improved autophagy, and improved behavioral outcomes (136, 137). Representative animal evidence is summarized in (**Table 4**). These findings should be interpreted as mechanism-generating rather than efficacy-confirming evidence, because most studies were conducted in transgenic or injection-based mouse models and have not been validated in rigorous AD clinical trials (133, 134, 136, 137, 140).

**Table 4:** Representative *in vivo* preclinical evidence for natural products and traditional Chinese medicine formulas targeting microglial networks in Alzheimer’s disease.

Representative *in vivo* preclinical evidence for natural products and traditional Chinese medicine formulas targeting microglial networks in Alzheimer’s disease					
Therapeutic agent	Preclinical model	Route and dosing regimen	Key microglia-related mechanisms	Main phenotypic/behavioral outcomes	Reference(s)
Curcumin	Amyloid-beta1–42 hippocampal injection mouse model	Oral gavage, 50 mg/kg/day for 8 weeks	Suppressed inflammatory signaling and oxidative stress and activated AMPK-associated pathways.	Improved spatial memory and reduced neuronal injury.	(138)
Berbamine	APP/PS1 transgenic mice	Intraperitoneal injection, 15 mg/kg/day	Bound the FKBP12-rapamycin-binding domain of mTOR and relieved repression of autophagy initiation.	Restored autophagic-lysosomal flux and accelerated hippocampal amyloid-beta clearance.	(132)
Resveratrol	APP/PS1 transgenic mice	Drinking water supplementation or oral gavage, 30–50 mg/kg/day	Reduced inflammatory cytokines, improved mitochondrial quality control, and modulated Nrf2/ARE and PI3K/Akt/mTORC1 signaling.	Partially improved long-term spatial memory.	(123)
Liuwei Dihuang Pill	APP/PS1 transgenic mice	Oral gavage, clinically equivalent dose for 12 weeks	Restored suppressed PI3K/Akt survival signaling and rebalanced pro-inflammatory and anti-inflammatory mediators.	Reduced toxic astroglial and microglial activation and lowered hippocampal neuronal apoptosis.	(136)
Yishen Huazhuo decoction	APP/PS1 transgenic mice	Oral gavage, 18.5 g/kg/day	Upregulated protective TREM2 expression and reshaped the TREM2/NF-κB axis.	Promoted a phagocytic, inflammation-resolving microglial phenotype, reduced cortical plaque burden, and improved working memory.	(137)
Cycloastragenol	5 × FAD transgenic mice (9-month-old)	Oral gavage (dose not specified in the original study)	Enhanced microglial phagocytosis; reduced microglial senescence; PDE4B/CREB/BDNF pathway identified as a downstream regulatory mechanism	Ameliorated cognitive impairments; reduced hippocampal Aβ deposition; alleviated microglial senescence	(139)

This table highlights representative animal studies relevant to the review theme and is not intended to be exhaustive. Dosing schedules are reported as described in the cited studies. All evidence is derived from preclinical models and has not been validated in rigorous AD clinical trials. AD, Alzheimer’s disease; AMPK, AMP-activated protein kinase; APP, amyloid precursor protein; ARE, antioxidant response element; Aβ, amyloid-beta; FKBP12, FK506-binding protein 12; mTOR, mechanistic target of rapamycin; NF-κB, nuclear factor kappa B; Nrf2, nuclear factor erythroid 2-related factor 2; PI3K, phosphoinositide 3-kinase; PS1, presenilin 1; *TREM2*, triggering receptor expressed on myeloid cells 2.

Clinical translation faces major barriers, including low oral bioavailability, limited brain exposure, poor pharmacokinetic stability, uncertain blood-brain barrier penetration, and lack of cell-type specificity (133–135, 141–144). To address these challenges, biomimetic nanodelivery systems have been proposed to improve BBB penetration, lesion-site accumulation, microglial uptake, and stimuli-responsive release (141–148). Carriers modified with transferrin receptor ligands, peptides, macrophage or microglial membrane camouflage, mannose ligands, ROS-sensitive linkers, or acid-sensitive bonds may improve delivery in experimental systems (142, 145–148).

Despite advances in experimental nanodelivery systems, Alzheimer’s nanomedicine is not ready for clinical use. First, CNS toxicity must be thoroughly checked. Nanoparticles may trigger immune responses, alter blood vessel behavior, accumulate around vessels, enter microglia by accident, or disrupt cellular waste clearance (142–144, 146). A carrier meant to reduce inflammation could unexpectedly cause it. Second, most data on Alzheimer’s nanomedicine come from lab studies, and few systems show consistent results in humans, proven microglial targeting, or real clinical benefits (141–148). Third, making and controlling these platforms is hard. The size, charge, coating, drug amount, release rate, cleanliness, stability, and batch consistency can all affect how nanoparticles travel through the body and are recognized by the body. Fourth, using release mechanisms that respond to oxidation or acidity needs caution. The stresses these mechanisms target are not limited to diseased microglia—they are found in other brain cells and stressed tissues too. So, stimulus-targeting may be less specific in people than in lab models (142–144, 146–148).

These limitations highlight another issue: bystander uptake. Mannose receptors, scavenger receptors, Fc receptors, and adhesion receptors are found not only in microglia but also in astrocytes, endothelial cells, perivascular macrophages, and peripheral monocytes, which can also take up carriers during inflammation (142, 145, 146). To confirm microglia specificity, nanomedicine studies should include human BBB models, iPSC-derived microglia, vascularized organoids or assembloids, aged animal models, biodistribution studies, chronic toxicity tests, and immune activation profiling before making claims (75, 76, 141–148).

A second challenge in using multicomponent natural or traditional formulas is the presence of pharmacological noise. These formulas may have many compounds, but only a few enter the bloodstream and even fewer reach the brain (133–135, 140). While this complexity supports the multi-target approach, it makes claims of “precise calibration” hard unless active ingredients, their amounts, and target effects are clearly defined (12, 13, 42–44, 133–135). Future research should measure chemical composition, identify key ingredients, track how compounds move through the body and brain, detect metabolites, test blood-brain barrier permeability, assess interactions, and use standardized safety testing. Herb-drug interactions are important if these formulas are taken with other medications, such as anti-Aβ antibodies, anticoagulants, antiplatelet agents, antidiabetic drugs, or immunomodulators (133–135). **Supplementary Table 3** lists the evidence, unknowns in drug behavior, standardization issues, risks of interactions, and regulatory obstacles for key natural products and TCM formulas.

## 6 Clinical translation: from anti-Aβ monoclonal antibodies to multi-targeted ‘escort’ strategies

Lecanemab and donanemab mark genuine clinical milestones, demonstrating that amyloid reduction yields statistically significant disease modification in early symptomatic AD (8, 9). This discussion aims to clarify the biological conditions that maximize the effectiveness and safety of amyloid clearance. While amyloid removal is clinically meaningful, its modest cognitive benefit and amyloid-related imaging abnormalities suggest it alone may not restore neuroimmune, synaptic, metabolic, or vascular homeostasis (8–10, 149).

Antibody-mediated amyloid clearance is not an isolated biochemical event. It alters vascular amyloid burden, complement activation, Fc receptor engagement, microglial activation, perivascular immune responses, and blood-brain barrier integrity (8–10, 149, 150). In susceptible patients, especially APOE ϵ4 carriers or individuals with cerebral amyloid angiopathy, rapid amyloid mobilization may destabilize the neurovascular unit and contribute to ARIA-E or ARIA-H (8–10, 149).

### 6.1 Perivascular macrophages and neurovascular immune instability during antibody treatment

ARIA is not solely a consequence of amyloid extraction from the parenchyma. The perivascular compartment acts as a transitional site where vascular amyloid, antibody–Aβ complexes, complement cascades, and myeloid cell activity converge (149, 150). Perivascular macrophages, positioned at the junction of the vascular wall, drainage pathways, and the blood-brain barrier, orchestrate immune mediation and promote amyloid removal within this domain (150). Anti-Aβ antibody therapy may induce accumulation of antibody-tagged vascular or perivascular amyloid in this niche prior to its full clearance.

If the load of antibody-opsonized Aβ exceeds the phagocytic and trafficking capacity of perivascular macrophages, Fc receptor engagement, complement activation, endothelial stress, basement membrane disruption, and increased vascular permeability may occur (149, 150). Parenchymal microglia may then respond secondarily to vascular leakage, complement fragments, cytokines, and signals from damaged tissue (150, 151). This model does not exclude microglial involvement; rather, it places microglia within a broader perivascular immune cascade. Therapeutically, it suggests that adjunctive strategies should not only suppress excessive microglial inflammation but also preserve BBB integrity, support perivascular clearance, stabilize endothelial responses, and prevent excessive complement activation around cerebral vessels (149–151).

**Figure 2:**
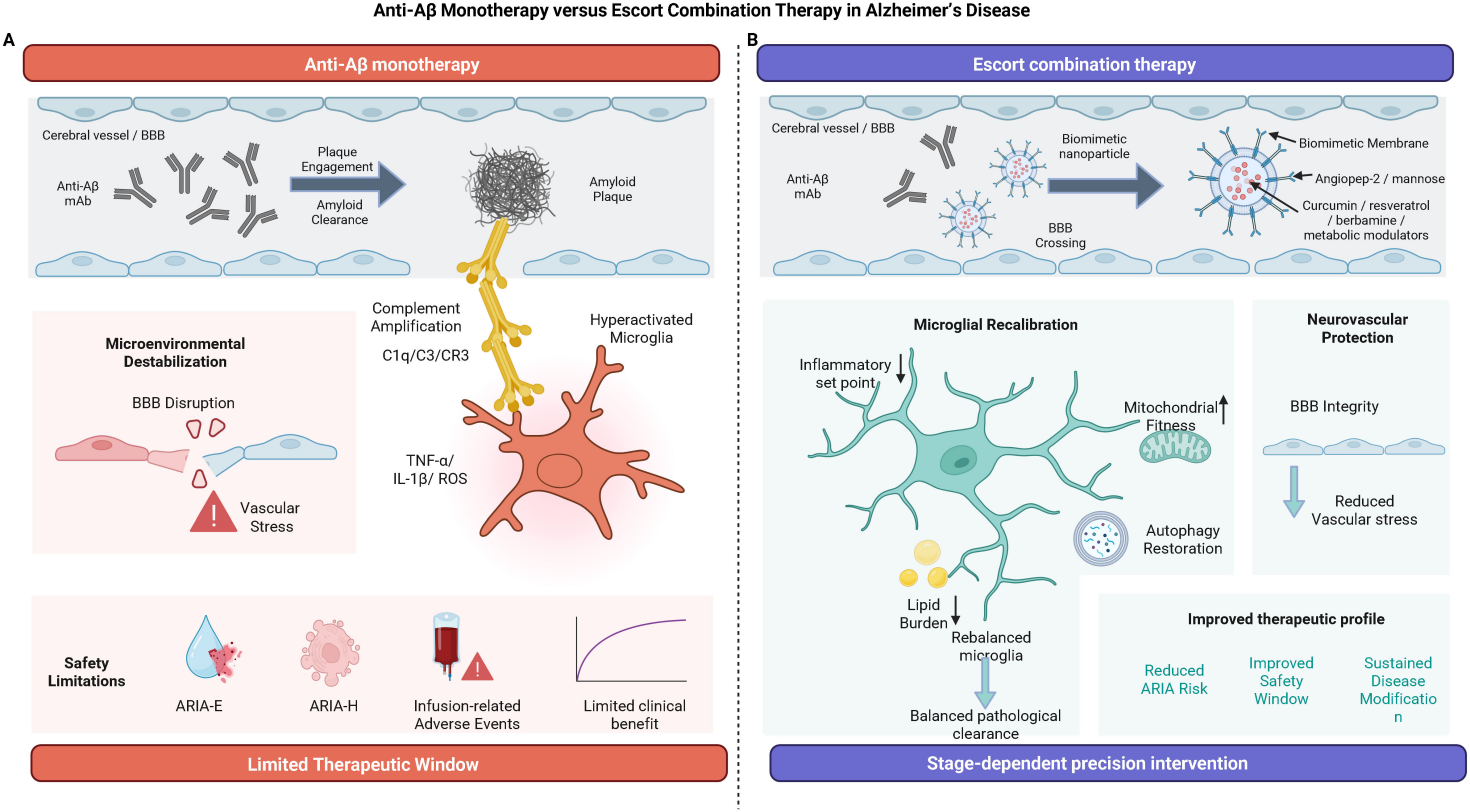
Anti-Aβ monotherapy versus escort combination therapy in Alzheimer’s disease. **(A)** The left panel summarizes the limitations of anti-Aβ monoclonal antibody (mAb) monotherapy. Following plaque engagement and amyloid clearance, rapid pathological mobilization may destabilize the local microenvironment, amplify complement signaling through the C1q/C3/CR3 axis, overactivate microglia, and increase production of TNF-α, IL-1β, and ROS. These changes may exacerbate vascular stress, disrupt the blood–brain barrier (BBB), and contribute to safety limitations, including amyloid-related imaging abnormalities-edema/effusion (ARIA-E), amyloid-related imaging abnormalities-hemorrhage/hemosiderin deposition (ARIA-H), infusion-related adverse events, and a restricted therapeutic window with limited clinical benefit. **(B)** The right panel illustrates the proposed escort combination therapy framework. In this model, anti-Aβ mAbs are combined with biomimetic nanoparticles engineered for BBB crossing and microglial targeting through a biomimetic membrane and ligands such as angiopep-2 and mannose. Therapeutic candidate therapeutic cargoes, including curcumin, resveratrol, berbamine, and metabolic modulators, are delivered in a stage-dependent precision intervention paradigm to recalibrate microglial states. This strategy is intended to lower the inflammatory set point, reduce lipid burden, restore autophagy, improve mitochondrial fitness, preserve neurovascular protection and BBB integrity, reduce vascular stress, and ultimately achieve balanced pathological clearance with reduced ARIA risk, a wider safety window, and more sustained disease modification. Aβ, amyloid-beta;ARIA, amyloid-related imaging abnormalities;ARIA-E, amyloid-related imaging abnormalities-edema/effusion;ARIA-H, amyloid-related imaging abnormalities-hemorrhage/hemosiderin deposition;BBB, blood–brain barrier;C1q, complement component 1q;C3, complement component 3;CR3, complement receptor 3;IL-1β, interleukin-1 beta;mAb, monoclonal antibody;ROS, reactive oxygen species;TNF-α, tumor necrosis factor alpha.

**Figure 2** compares conventional anti-Aβ monotherapy with the proposed escort combination therapy. It highlights microenvironmental stabilization and improved safety.

### 6.2 Escort-style combination therapy

The “escort” strategy does not aim to impair antibody-mediated clearance of pathology. Antibodies remain the primary mechanism for pathology clearance, whereas adjunctive interventions stabilize the immune-metabolic-vascular microenvironment (12, 42, 43). Escort interventions may reduce excessive microglial and perivascular macrophage activation, improve metabolic resilience, support lysosomal and mitochondrial function, raise pathological synaptic pruning thresholds, protect the BBB and neurovascular unit, and reduce ARIA risk while preserving amyloid clearance (8–10, 117, 149, 150). Potential escort agents include metabolic modulators such as metformin (41, 107, 108, 119, 120), mitochondrial protectants such as coenzyme Q10 (113, 114), carefully timed autophagy modulators such as rapamycin-like strategies (70–72, 112, 115, 116), pro-resolving anti-inflammatory agents (129–131), and nanodelivered low-intensity natural bioactive molecules (141–148).

### 6.3 Biomarker-guided calibration of escort therapy

A practical escort strategy demands biomarker-driven calibration. Empirical combination therapy is inadequate. Patients require tailored adjunctive modules, selected based on the dominant microenvironmental instability (31, 73, 117, 118). For example, patients with high amyloid burden, APOE ϵ4 genotype, cerebral amyloid angiopathy, or baseline microbleeds may benefit from vascular-protective and complement-buffering strategies during antibody initiation (8–10, 149, 150). Those with elevated inflammatory markers, high CSF sTREM2, or increased glial activation signals may need low-intensity immunomodulatory support (73, 117). Individuals with metabolic dysfunction, insulin resistance, mitochondrial injury, or lipid dysregulation may be better suited for metabolic or lysosome-supporting interventions (37–41, 69–74, 103–111, 113, 114, 117).

Potential biomarker panels should integrate pathology, glial activation, neuronal injury, and vascular risk factors. Amyloid PET and plasma or CSF Aβ42/40 precisely define the amyloid clearance target. Tau PET or phosphorylated tau species pinpoint downstream neurodegenerative burden. CSF sTREM2 signals microglial activation alongside phagocytic engagement. GFAP marks astroglial and neurovascular stress (73, 117). NfL delivers a nonspecific yet informative readout of ongoing neuroaxonal injury. Complement fragments, inflammatory cytokines, lipidomic markers, and MRI evidence of microbleeds or superficial siderosis sharpen risk stratification (117, 149).

The endpoint of escort therapy should not be maximal suppression of inflammatory biomarkers. Instead, therapy goals should be explicitly defined as amyloid reduction, stable BBB integrity, absence or reduction of ARIA, preservation of synaptic and neuronal injury markers, and maintenance of adaptive microglial responses (117, 149). This approach shifts clinical development from a pathology-only model to an integrated model of pathology clearance plus microenvironment protection.

### 6.4 Operationalizing microglial state-calibration in clinical trials

Microglial state-calibration is mechanistically attractive, but without trial-ready criteria, it remains conceptual. A clinically useful approach must clarify three elements: baseline state assignment, intervention selection, and evidence of successful recalibration (31, 117). Baseline assignment should integrate multiple biomarkers rather than a single marker. These may include amyloid and tau burden, combined with fluid and imaging biomarkers for microglial activation, astrocytic response, axonal injury, complement activation, lipid stress, metabolic dysfunction, and vascular risk (31, 73, 117, 118, 149). Example markers are CSF or plasma sTREM2, GFAP, NfL, inflammatory cytokines, complement fragments, lipidomic signatures, lactate-related metabolic readouts, mitochondrial injury markers, amyloid PET, tau PET, and vascular MRI findings—such as microbleeds, superficial siderosis, white matter hyperintensities, CAA-related markers—and neuroinflammation-sensitive imaging (31, 73, 117, 118, 149).

A practical trial design could stratify participants into biologically enriched subgroups. For example, patients with high amyloid burden but modest inflammatory and vascular injury markers may be suitable for amyloid-directed therapy alone or with low-intensity metabolic support. Patients with elevated sTREM2, GFAP, complement activation, lipid dysregulation, APOE ϵ4-associated vascular risk, or baseline microbleeds may require an escort strategy. This strategy combines pathology clearance with immune-metabolic or neurovascular stabilization (31, 117, 149). These thresholds are not yet validated and should be treated as exploratory enrichment variables rather than definitive clinical decision rules.

Successful recalibration should use composite endpoints. Cognitive scales remain essential. Surrogate endpoints may include amyloid reduction with stable BBB integrity, reduced or absent ARIA, stabilization of GFAP and NfL, decreased complement activation, improved lipidomic profiles, reduced vascular injury signals, and preservation of synaptic or network integrity (117, 118, 149). In this framework, success is not maximal suppression of inflammation. Instead, it means restoring adaptive immune-metabolic function while minimizing collateral synaptic and vascular injury.

(**Table 5**) proposes an exploratory translational framework based on this logic. It links dominant microglial states to candidate biomarker suites, therapeutic strategies, and operational endpoints for successful calibration. This framework aims to guide hypothesis generation and biomarker-enriched trial design. It does not provide validated clinical decision thresholds.

**Table 5:** Proposed translational framework for microglial state-calibration.

Proposed translational framework for microglial state-calibration				
Target microglial state	Suitable biomarker suite (CSF/imaging)	Therapeutic strategy	Endpoint for successful calibration	Reference(s)
Early plaque-associated DAM with preserved phagocytosis	Amyloid PET; CSF/plasma Aβ42/40; CSF sTREM2; TSPO PET if available	Mild TREM2-pathway support; lipid-homeostasis modulation; cautious antibody clearance	Plaque reduction with stable or moderately increased sTREM2, no excessive inflammatory biomarker rise	(31, 58, 59, 78–80, 117)
Lipid-overloaded *APOE* ϵ4-associated microglia	*APOE* genotype; lipidomic markers; CSF sTREM2; GFAP; amyloid/vascular imaging	LXR/ABCA1-related lipid efflux support; metabolic modulators; mitochondrial protection	Improved lipid-handling signatures, preserved cognition, reduced inflammatory activation	(29, 32, 69, 70, 73, 95, 117)
Complement-pruning dominant microglia	Synaptic PET or synaptic-density imaging if available and validated; CSF complement markers C1q/C3 fragments; NfL; cognitive network measures	Complement-threshold modulation; CD47–SIRPα support; synapse-protective strategies	Reduced synaptic injury biomarkers and NfL without impaired debris clearance	(33–36, 82–85, 118)
Glycolytic/inflammatory microglia	CSF cytokines; CSF/plasma lactate-related metabolites, NAD+ pathway metabolites, or other exploratory metabolic readouts; TSPO PET; GFAP; NfL	AMPK activation; HIF-1α/PKM2 modulation; anti-inflammatory resolution agents	Reduced inflammatory tone with preserved phagocytic markers and metabolic flexibility	(37–41, 101, 102, 106, 117)
LDAM/senescent microglia	*APOE* ϵ4 status; lipid droplet-related signatures; SASP markers; NfL; MRI atrophy	Restore lysosomal function first; then cautious autophagy induction; rapamycin/metformin-like strategies	Improved autophagic flux, reduced SASP markers, stabilized neurodegeneration biomarkers	(29, 69–72, 112, 117)
Interferon-responsive microglia	IFN-stimulated gene signatures; *CXCL10*; *STING*-related markers; mitochondrial injury markers	Mitophagy enhancement; cGAS–STING pathway modulation; mitochondrial repair	Reduced interferon signature without broad immunosuppression	(23, 27, 86–91, 117)
Antibody-treatment vulnerable perivascular immune state	*APOE* genotype; CAA imaging markers; microbleeds; vascular MRI; inflammatory markers	Escort therapy during antibody treatment; PVM/microglia inflammatory buffering; vascular protection	Amyloid reduction with lower ARIA frequency and preserved BBB integrity	(8–10, 149–151)

This framework is intended to guide hypothesis generation and biomarker-enriched trial design rather than to provide validated clinical decision thresholds. All biomarker suites and therapeutic strategies are exploratory and require prospective validation.

ABCA1, ATP-binding cassette transporter A1; AD, Alzheimer’s disease; AMPK, AMP-activated protein kinase; APOE, apolipoprotein E; ARIA, amyloid-related imaging abnormalities; Aβ, amyloid-beta; BBB, blood–brain barrier; C1q, complement component 1q; C3, complement component 3; CAA, cerebral amyloid angiopathy; CD47, cluster of differentiation 47; cGAS, cyclic GMP-AMP synthase; CSF, cerebrospinal fluid; CXCL10, C-X-C motif chemokine ligand 10; DAM, disease-associated microglia; GFAP, glial fibrillary acidic protein; HIF-1α, hypoxia-inducible factor 1 subunit alpha; IFN, interferon; IRM, interferon-responsive microglia; LDAM, lipid droplet-accumulating microglia; LXR, liver X receptor; MRI, magnetic resonance imaging; mTOR, mechanistic target of rapamycin; NAD^+^, nicotinamide adenine dinucleotide; NfL, neurofilament light chain; PET, positron emission tomography; PKM2, pyruvate kinase M2; PVM, perivascular macrophage; SASP, senescence-associated secretory phenotype; SIRPα, signal regulatory protein alpha; STING, stimulator of interferon genes; sTREM2, soluble triggering receptor expressed on myeloid cells 2; *TREM2*, triggering receptor expressed on myeloid cells 2; TSPO, translocator protein.

### 6.5 Genetic stratification: promise and feasibility limits

Genetic stratification is important but hard to do. APOE ϵ4, TREM2, PLCG2, and ABCA7 can affect inflammation, cell clearance, lipid handling, vascular risk, and ARIA risk. Future studies should combine gene types with markers like CSF sTREM2, GFAP, NfL, cytokines, complement, fat patterns, and imaging findings (31, 73, 117, 118, 149).

Making different carriers or treatments for each gene type is not practical. A better way is a basic core with a changeable targeting part. The main carrier provides steady drug levels, brain access, and safety. Changeable surface markers or dose plans can be tailored to each subgroup (141–148). This method allows accuracy without complex production.

For example, APOE ϵ4 carriers with lipid-handling defects may benefit from adjunctive strategies that promote cholesterol efflux, lysosomal support, and vascular protection, rather than simply stronger microglial activation (29, 32, 69, 73, 95, 117). Patients with TREM2 or PLCG2-associated differences in immune response may require different thresholds for immune stimulation or anti-inflammatory escort therapy (15, 18, 87, 92–94, 97, 98, 100, 124). Such genotype-guided approaches remain hypothetical and should initially be tested as stratified exploratory analyses rather than as separate commercial products. The realistic near-term goal is not genotype-specific nanomedicine for every patient, but biomarker-informed adjustment of treatment intensity, timing, and adjunctive support (31, 73, 117).

## 7 Conclusions and future prospects

Microglia are not passive in AD. They act as regulatory hubs, effectively connecting Aβ deposition, tau pathology, synaptic loss, lipid imbalance, mitochondrial dysfunction, autophagy failure, neurovascular stress, and chronic inflammation. Single-cell and spatial omics reveal microglia shift through continuous, flexible, and context-dependent states, opening new opportunities for understanding and intervention.

Mechanistically, the TREM2–APOE axis positively influences microglial activation and survival, while the complement cascade facilitates synapse remodeling. Immunometabolic reprogramming enables adaptive energy use and balanced inflammatory responses. Although challenges such as impaired lipid handling, complement overactivation, chronic glycolysis, and mitochondrial damage exist, ongoing discoveries around mtDNA-cGAS-STING signaling offer promising avenues to link mitochondrial injury to interferon-responsive microglial states (37–41, 69–74, 88–91, 101–111, 117).

Anti-Aβ antibodies demonstrate that lowering amyloid can positively influence disease progression. While pathology clearance alone may not always restore brain homeostasis (8–10, 149), future therapies have the exciting potential to combine pathology clearance with microenvironment protection. This promising approach could stabilize microglial states, safeguard synapses, enhance metabolic flexibility, reduce neurovascular stress, and be tailored to disease stage and host background (31, 73, 117, 149).

As outlined in the proposed framework above, biomarker-enriched and stage-specific trial designs can drive more tailored and successful microglial state-calibration, moving beyond uniform anti-inflammatory strategies and offering hope for precision medicine in AD. In summary, integrating microglial state-calibration into therapeutic development holds promise for advancing disease-modifying treatments and achieving lasting brain homeostasis in AD.

### 7.1 Future research priorities

Several priorities should guide future research. First, microglial states should be studied spatially. Bulk inflammatory markers cannot distinguish plaque-proximal DAM, distal aging microglia, interferon-responsive microglia, lipid-overloaded microglia, and perivascular myeloid cells. Spatial transcriptomics, proteomics, and imaging biomarkers should therefore be integrated to define where and when intervention is most appropriate (25, 75, 118).

Make human validation central. Use human iPSC-derived microglia, chimeric models, spatial omics, and biomarker studies to bridge the translation gap. Consideration of sex as a biological variable further advances research precision (23, 25–31, 75–77, 117).

Treat sex as a biological variable. Report sex-disaggregated outcomes, analyze sex-genotype interactions, and ensure clinical trials use sex stratification. Sequencing combination therapies is another cornerstone of future research.

Optimize sequence for combination therapies. In lipid-overloaded microglia, restore degradation and efflux before autophagy. Use adaptive trial designs with biomarker endpoints. Together, these priorities converge toward recalibrating microglial states as a therapeutic strategy (8–10, 149, 150).

Microglial state recalibration is therefore more than a refinement of anti-inflammatory therapy. It represents a shift from suppressing disease signals to restoring functional homeostasis. The main objective is to achieve effective therapies that combine patient stratification, pathology clearance, immune-metabolic regulation, synaptic protection, vascular stabilization, and smart delivery (12, 31, 42, 43, 117, 141–148). Through this approach, the intended impact is to transition microglia from maladaptive disease-amplifying states to protective, adaptive, and tissue-supportive roles.
