# Supplementary material for: Calibrating microglia states in Alzheimer’s disease: decoding immune-metabolic networks and nano-targeted multicomponent therapies
Source: Front Immunol. 2026 Jun 16;17:1843978. doi: 10.3389/fimmu.2026.1843978 (PMC13314446; doi:10.3389/fimmu.2026.1843978)
Supplement: Supplementary file 1 [file DataSheet1.pdf]

Supplementary Table 1. Translational Certainty Assessment of Key Mechanistic Claims

Note: Evidence categories are defined as follows: Strong = multiple independent studies with consistent results; Moderate = supporting evidence with some inconsistency or limited direct replication; Limited = sparse, indirect, or primarily qualitative evidence; Emerging = actively developing evidence base with recent preliminary findings.AD, Alzheimer's disease; AMPK, AMP-activated protein kinase; Aβ, amyloid-beta; BBB, blood–brain barrier; c*GAS*, cyclic GMP-AMP synthase; CMC, chemistry, manufacturing, and controls; CNS, central nervous system; CSF, cerebrospinal fluid; GFAP, glial fibrillary acidic protein; iPSC, induced pluripotent stem cell; IRM, interferon-responsive microglia; LDAM, lipid droplet-accumulating microglia; MRI, magnetic resonance imaging; mTOR, mechanistic target of rapamycin; NfL, neurofilament light chain; PET, positron emission tomography; PVM, perivascular macrophage; ROS, reactive oxygen species; *STING*, stimulator of interferon genes; sTREM2, soluble triggering receptor expressed on myeloid cells 2; TCM, traditional Chinese medicine; TSPO, translocator protein; *ULK1*, Unc-51 like autophagy activating kinase 1.

Translational Certainty Assessment of Key Mechanistic Claims							
**Key mechanistic claim**	**Mouse / animal model evidence**	**Human post-mortem evidence**	**iPSC-derived or humanized microglia evidence**	**Clinical biomarker / trial evidence**	**Translational certainty to sporadic human AD**	**Comment**	**Reference(s)**
Chronic glycolytic reprogramming drives inflammatory microglial dysfunction	Strong	Moderate transcriptomic association	Moderate	Limited	Medium	Immunometabolic remodeling is well supported, but direct causal proof in sporadic human AD is incomplete.	(1–8)
Mitochondrial dysfunction and ROS	Strong	Moderate	Moderate	Limited	Medium	Strong mechanistic plausibility; human	(8–14)

Translational Certainty Assessment of Key Mechanistic Claims							
**Key mechanistic claim**	**Mouse / animal model evidence**	**Human post-mortem evidence**	**iPSC-derived or humanized microglia evidence**	**Clinical biomarker / trial evidence**	**Translational certainty to sporadic human AD**	**Comment**	**Reference(s)**
promote LDAM/exhausted microglial states						evidence is mainly associative through transcriptomic, lipidomic, and aging-related signatures.	
mtDNA leakage activates c*GAS–STING* and contributes to interferon-responsive microglia	Moderate in cell and animal systems	Emerging	Limited to moderate	Limited	Low to medium	Mechanistically compelling and linked to IRM-like signatures, but direct validation in human sporadic AD remains developing.	(15–18)
AMPK–mTOR–*ULK1* imbalance impairs autophagy and metabolic resilience in AD microglia	Moderate to strong	Limited to moderate	Limited	Emerging from metabolic intervention studies	Medium	Supported by mechanistic biology and drug studies; microglia-specific human evidence remains limited.	(19–26)
Natural products such as curcumin, berberine,	Moderate preclinical	Limited	Limited	Limited for selected	Low to medium	Pleiotropic mechanisms are	(27–29)

Translational Certainty Assessment of Key Mechanistic Claims							
**Key mechanistic claim**	**Mouse / animal model evidence**	**Human post-mortem evidence**	**iPSC-derived or humanized microglia evidence**	**Clinical biomarker / trial evidence**	**Translational certainty to sporadic human AD**	**Comment**	**Reference(s)**
and resveratrol recalibrate microglial networks				agents		plausible, but bioavailability, dosing, CNS exposure, reproducibility, and clinical efficacy remain major barriers.	
TCM formulas modulate microglial inflammatory and metabolic networks	Moderate preclinical	Very limited	Very limited	No definitive AD clinical trial evidence for most formulas	Low	Evidence is largely mechanism-generating. Standardization, pharmacological noise, PK uncertainty, herb-drug interactions, and regulatory requirements remain substantial barriers.	(30, 31)
Biomimetic nanodelivery improves brain exposure and microglial targeting	Strong preclinical engineering rationale	Limited	Limited	Very limited in AD	Low	Promising but clinically immature. CNS toxicity, off-target uptake, CMC, human BBB penetration, and	(32–39)

Translational Certainty Assessment of Key Mechanistic Claims							
**Key mechanistic claim**	**Mouse / animal model evidence**	**Human post-mortem evidence**	**iPSC-derived or humanized microglia evidence**	**Clinical biomarker / trial evidence**	**Translational certainty to sporadic human AD**	**Comment**	**Reference(s)**
						stimulus-responsive release require validation.	
Anti-Aβ antibody adverse events reflect difficulty stabilizing neuroimmune and neurovascular microenvironments during rapid clearance	Limited animal and mechanistic inference	Moderate vascular amyloid association	Limited	Strong clinical ARIA evidence	Medium	ARIA is clinically established. Mechanistic roles of PVMs, microglia, complement, Fc receptors, and BBB stress require further validation.	(40–45)
Microglial state calibration can be operationalized using biomarker-guided stratification	Limited	Limited	Limited	Emerging biomarker evidence	Low to medium	The framework remains theoretical. Candidate biomarkers include sTREM2, GFAP, NfL, cytokines, complement markers, lipidomic signatures, TSPO PET, amyloid/tau PET, and vascular MRI.	(8, 46–48)

Supplementary Table 2.Representative clinical development programs targeting microglial signaling, neuroinflammation, immunometabolism, and related pathways in Alzheimer's disease

Note: Clinical study titles are reported in American English and standardized for consistency. Trial phases and recruitment status are reproduced from the source material. AAV, adeno-associated virus; *ABCA1*, ATP-binding cassette transporter A1; AD, Alzheimer's disease; aMCI, amnestic mild cognitive impairment; AMPK, AMP-activated protein kinase; APOE, apolipoprotein E; ARIA, amyloid-related imaging abnormalities; Aβ, amyloid-beta; c-Abl, Abelson tyrosine kinase; CCL2, C-C motif chemokine ligand 2; *CD33*, cluster of differentiation 33; CD38, cluster of differentiation 38; c-Kit, receptor tyrosine kinase KIT; CSF, cerebrospinal fluid; CSF-1R, colony-stimulating factor 1 receptor; GFAP, glial fibrillary acidic protein; GLP-1, glucagon-like peptide 1; GM-CSF, granulocyte-macrophage colony-stimulating factor; HDL, high-density lipoprotein; IL-1β, interleukin-1 beta; JAK, Janus kinase; *MAPT*, microtubule-associated protein tau; MCI, mild cognitive impairment; MSC-Exos, mesenchymal stem cell-derived exosomes; mTOR, mechanistic target of rapamycin; NF-κB, nuclear factor kappa B; NfL, neurofilament light chain; PGRN, progranulin; PKR, protein kinase R; *SORT1*, sortilin 1; sTREM2, soluble triggering receptor expressed on myeloid cells 2; TREM2, triggering receptor expressed on myeloid cells 2.

**Representative clinical development programs targeting microglial signaling, neuroinflammation, immunometabolism, and related pathways in Alzheimer's disease**										
**Category**	**Target**	**Agent**	**Mechanism / modality**	**Trial ID**	**Official study title**	**Population**	**Phase**	**Status**	**Key findings / remarks**	**Referenc e(s)**
Receptor	TREM2	AL002	Agonistic monoclonal antibody	NCT0363 5047	A Phase I Study of AL002 in Healthy Volunteers and	Healthy volunteers; AD	Phase I	Comple ted	Generally safe and well tolerated; increased CSF sTREM2.	(49, 50)

Representative clinical development programs targeting microglial signaling, neuroinflammation, immunometabolism, and related pathways in Alzheimer's disease										
**Category**	**Target**	**Agent**	**Mechanism / modality**	**Trial ID**	**Official study title**	**Population**	**Phase**	**Status**	**Key findings / remarks**	**Referenc e(s)**
					Participants With Mild-to-Moderate Alzheimer's Disease					
Receptor	TREM2	AL002	Agonistic monoclonal antibody	NCT04592874	A Phase 2 Study to Evaluate Efficacy and Safety of AL002 in Participants With Early Alzheimer's Disease (INVOKE-2)	Early AD	Phase II	Completed	Did not meet the primary endpoint; ARIA was among the most common adverse events; biomarker focus included sTREM2.	(51)
Receptor	TREM2	AL002	Agonistic monoclonal	NCT05744401	A Long-term Extension	AD	Phase II	Terminated	Long-term extension was	(52)

Representative clinical development programs targeting microglial signaling, neuroinflammation, immunometabolism, and related pathways in Alzheimer's disease										
**Category**	**Target**	**Agent**	**Mechanism / modality**	**Trial ID**	**Official study title**	**Population**	**Phase**	**Status**	**Key findings / remarks**	**Reference(s)**
			antibody		Study to Evaluate the Safety, Tolerability, and Efficacy of AL002 in Participants With Alzheimer's Disease		extension		stopped after the parent study was unsuccessful.	
Receptor	TREM2	VG-3927	Small-molecule agonist	NCT06343636	A Phase 1 Study of VG-3927 in Healthy Adults and Patients With Alzheimer's Disease	Healthy adults; AD	Phase I	Completed	Designed to assess safety and target engagement; biomarkers included CSF sTREM2.	(53)
Receptor	*CD33*	AL003	Antagonistic	NCT0382	First in Human	AD	Phase	Comple	Primarily	(54)

Representative clinical development programs targeting microglial signaling, neuroinflammation, immunometabolism, and related pathways in Alzheimer's disease										
**Category**	**Target**	**Agent**	**Mechanism / modality**	**Trial ID**	**Official study title**	**Population**	**Phase**	**Status**	**Key findings / remarks**	**Reference(s)**
			monoclonal antibody	2208	Study for Safety and Tolerability of AL003		I	ted	evaluated safety; exploratory biomarkers included peripheral monocyte *CD33* expression.	
Signaling pathway	AMPK	Metformin	AMPK activator / insulin sensitizer	NCT01965756	Effect of Insulin Sensitizer Metformin on AD Biomarkers	AD	Phase II	Completed	Reported symptomatic cognitive benefit with an acceptable safety profile.	(55, 56)
Signaling pathway	AMPK	Metformin	AMPK activation with indirect NF-κB modulation	NCT04098666	Metformin in Alzheimer's Dementia Prevention (MAP)	AD prevention	Phase II/III	Ongoing	Large prospective prevention trial; expected completion was listed for 2026 in the source material.	(57)

**Representative clinical development programs targeting microglial signaling, neuroinflammation, immunometabolism, and related pathways in Alzheimer's disease**										
**Category**	**Target**	**Agent**	**Mechanism / modality**	**Trial ID**	**Official study title**	**Population**	**Phase**	**Status**	**Key findings / remarks**	**Reference(s)**
Genetic risk pathway	*APOE*4	LX1001	AAV-mediated gene therapy	NCT03634007	Gene Therapy for *APOE*4 Homozygote of Alzheimer's Disease	*APOE*4 homozygous AD	Phase I/II	Ongoing	Interim data reported detectable APOE2 expression in CSF.	(58)
Genetic risk pathway	*APOE*4	CS6253	*ABCA1* agonist	NCT05965414	Safety and Pharmacokinetics of CS6253 in Healthy Volunteers	Healthy volunteers	Phase I	Completed	Showed favorable safety and increases in small HDL and ApoE in plasma.	(59)
Autophagy / kinase	mTOR	Rapamycin	mTOR inhibitor	NCT04200911	Cognition, Age, and RaPamycin Effectiveness - DownregulatIon of thE mTor Pathway (CARPE_DIE	MCI; AD	Phase I	Completed	CSF rapamycin was not directly detected; CSF p-tau181 increased after treatment in the reported dataset.	(26, 60)

Representative clinical development programs targeting microglial signaling, neuroinflammation, immunometabolism, and related pathways in Alzheimer's disease										
**Category**	**Target**	**Agent**	**Mechanism / modality**	**Trial ID**	**Official study title**	**Population**	**Phase**	**Status**	**Key findings / remarks**	**Reference(s)**
					M)					
Autophagy / kinase	mTOR	Rapamycin	mTOR inhibitor	NCT04629495	Rapamycin - Effects on Alzheimer's and Cognitive Health (REACH)	aMCI; early AD	Phase II	Ongoing	Evaluates the safety and feasibility of 12 months of oral rapamycin.	(61)
Autophagy / kinase	c-Abl/BCR-ABL	Nilotinib BE	Tyrosine kinase inhibitor	NCT05143528	Evaluating the Efficacy and Safety of Nilotinib BE in Subjects With Early Alzheimer's Disease (NILEAD)	Early AD	Phase III	Not yet recruiting	Designed to assess efficacy and amyloid-beta / tau biomarkers in early AD.	(62)
Autophagy / kinase	CSF-1R	EI-1071	Small-molecule inhibitor	NCT04238364	A Study to Evaluate the Safety,	Healthy volunteers	Phase I	Completed	Reported as safe and tolerated; positioned as a	(63)

Representative clinical development programs targeting microglial signaling, neuroinflammation, immunometabolism, and related pathways in Alzheimer's disease										
**Category**	**Target**	**Agent**	**Mechanism / modality**	**Trial ID**	**Official study title**	**Population**	**Phase**	**Status**	**Key findings / remarks**	**Reference(s)**
					Tolerability and Amount of EI-1071 in Healthy Volunteers				depletion-oriented strategy relevant to microglial exhaustion.	
Autophagy / kinase	c-Kit	Masitinib	Tyrosine kinase inhibitor	NCT05564169	Masitinib in Patients With Mild Alzheimer's Disease	Mild AD	Phase III	Ongoing	Evaluated as an add-on strategy to slow cognitive decline in mild AD.	(64)
Growth factor	GM-CSF	Sargramostim	Recombinant human GM-CSF	NCT01409915	Study of the Safety and Efficacy of Leukine in the Treatment of Alzheimer's Disease	AD	Phase II	Completed	Reported improvement in cognition and neurodegeneration-related biomarkers.	(65, 66)
Growth factor	GM-CSF	Sargramostim	Recombinant human	NCT04902703	Phase II Trial to Evaluate	AD	Phase II	Ongoing	Evaluates safety and changes in	(67)

Representative clinical development programs targeting microglial signaling, neuroinflammation, immunometabolism, and related pathways in Alzheimer's disease										
**Category**	**Target**	**Agent**	**Mechanism / modality**	**Trial ID**	**Official study title**	**Population**	**Phase**	**Status**	**Key findings / remarks**	**Reference(s)**
			GM-CSF		Safety and Efficacy of GM-CSF / Sargramostim in Alzheimer's Disease (SESAD)				plasma Aβ40, total tau, GFAP, and NfL.	
Immunomodulation	CD38	Daratumumab	Monoclonal antibody	NCT04070378	Study of Daratumumab in Patients With Mild to Moderate Alzheimer's Disease	Mild-to-moderate AD	Phase II	Completed	Reduced the proportion of CD38-positive cells without clear cognitive improvement.	(68, 69)
Immunomodulation	IL-1β	Canakinumab	IL-1β-neutralizing monoclonal antibody	NCT04795466	Study of the Efficacy and Safety of Various Anti-inflammat	MCI; mild AD	Phase II	Terminated	Designed to evaluate efficacy and safety of anti-inflammatory interventions.	(70)

Representative clinical development programs targeting microglial signaling, neuroinflammation, immunometabolism, and related pathways in Alzheimer's disease										
**Category**	**Target**	**Agent**	**Mechanism / modality**	**Trial ID**	**Official study title**	**Population**	**Phase**	**Status**	**Key findings / remarks**	**Referenc e(s)**
					ory Agents in Participants With Mild Cognitive Impairment or Mild Alzheimer's Disease					
Immunomodu lation	JAK	Baricitinib	JAK inhibitor	NCT0518 9106	Neurodegener ative Alzheimer's Disease and Amyotrophic Lateral Sclerosis (NADALS) Basket Trial	AD; ALS	Phase I/II	Ongoin g	Basket trial with biomarker endpoints including CSF CCL2, PKR, and NfL.	(71)
Neurotrophic / metabolic	GLP-1	Semaglutide	GLP-1 receptor	NCT0477 7396	A Research Study to Look	Early AD	Phase III	Ongoin g	Evaluates whether oral semaglutide	(72)

Representative clinical development programs targeting microglial signaling, neuroinflammation, immunometabolism, and related pathways in Alzheimer's disease										
**Category**	**Target**	**Agent**	**Mechanism / modality**	**Trial ID**	**Official study title**	**Population**	**Phase**	**Status**	**Key findings / remarks**	**Reference(s)**
			agonist		at How Well Semaglutide Slows Down Progression of Alzheimer's Disease (EVOKE)				slows cognitive decline in early AD.	
Lectin pathway	Galectin-3	TB006	Anti-Galectin-3 monoclonal antibody	NCT05074498	Study to Assess the Safety, Tolerability, Pharmacokinetics, Pharmacodynamics, and Efficacy of TB006 in Participants With Alzheimer's	AD	Phase I/II	Completed	Intended to dampen pro-inflammatory cascades; preliminary cognitive improvement was reported.	(73)

Representative clinical development programs targeting microglial signaling, neuroinflammation, immunometabolism, and related pathways in Alzheimer's disease										
**Category**	**Target**	**Agent**	**Mechanism / modality**	**Trial ID**	**Official study title**	**Population**	**Phase**	**Status**	**Key findings / remarks**	**Referenc e(s)**
					Disease					
Lectin pathway	Galectin-3	TB006	Anti-Galectin-3 monoclonal antibody	NCT0547 6783	A Long-Term Extension Study to Assess the Safety of TB006 in Participants With Alzheimer's Disease	AD	Phase II	Ongoin g	Assesses long-term safety of continued dosing.	(74)
Tau-directed	*MAPT* / tau	NIO752	Antisense oligonucleotid e	NCT0637 2821	A Trial Evaluating the Effect of NIO752 on Tau Synthesis Measured by a Process Known as	Early AD	Phase I	Ongoin g	Evaluates the effect of intrathecal NIO752 on tau synthesis rate.	(75)

Representative clinical development programs targeting microglial signaling, neuroinflammation, immunometabolism, and related pathways in Alzheimer's disease										
**Category**	**Target**	**Agent**	**Mechanism / modality**	**Trial ID**	**Official study title**	**Population**	**Phase**	**Status**	**Key findings / remarks**	**Reference(s)**
					SILK					
Natural product	Multitarget	Curcumin	Network-level modulation	NCT01811381	Curcumin and Yoga Therapy for Those at Risk for Alzheimer's Disease	At-risk population	Phase II	As reported in source	Developed to evaluate cognitive protection with curcumin-based combination intervention; bioavailability remains a translational challenge.	(76)
Natural product	Multitarget	Resveratrol	*SIRT1* activation	NCT01504854	Resveratrol for Alzheimer's Disease	AD	Phase II	Completed	Reduced CSF MMP9 and Aβ40 and was associated with slower disease progression signals.	(77, 78)
Natural	Multitarget	Resveratrol-con	SIRT1-linked	NCT0067	Randomized	AD	Phase	Comple	Low-dose oral	(79)

Representative clinical development programs targeting microglial signaling, neuroinflammation, immunometabolism, and related pathways in Alzheimer's disease										
**Category**	**Target**	**Agent**	**Mechanism / modality**	**Trial ID**	**Official study title**	**Population**	**Phase**	**Status**	**Key findings / remarks**	**Reference(s)**
product		taining nutritional supplement	pleiotropic modulation	8431	Trial of a Nutritional Supplement in Alzheimer's Disease		II	ted	treatment was safe and tolerated, with limited efficacy signals.	
Natural product	Multitarget	Trehalose	Autophagy-inducing strategy	NCT04663854	Mycose AdminiStration for HealIng Alzheimer NEuropathy (MASHIANE)	AD	Phase I	Ongoing	Evaluates safety and tolerability of trehalose administration.	(80)
Extracellular vesicle	Stem cell-derived exosomes	MSC-Exos	Allogeneic adipose mesenchymal stem cell exosomes	NCT04388982	The Safety and the Efficacy Evaluation of Allogenic Adipose MSC-Exos in Patients With	AD	Phase I/II	Unknown	Tests the cell-free anti-inflammatory potential of allogeneic adipose MSC-derived exosomes.	(81)

Representative clinical development programs targeting microglial signaling, neuroinflammation, immunometabolism, and related pathways in Alzheimer's disease										
**Category**	**Target**	**Agent**	**Mechanism / modality**	**Trial ID**	**Official study title**	**Population**	**Phase**	**Status**	**Key findings / remarks**	**Reference(s)**
					Alzheimer's Disease					
Sortilin pathway	sortilin *(SORT1)*	Nivisnebart (AL101)	Anti-SORT1 monoclonal antibody	NCT06079190	Efficacy and Safety of GSK4527226 [AL101] in Participants With Early Alzheimer's Disease (PROGRESS-AD)	Early AD	Phase II	Ongoing	Intended to increase progranulin by blocking sortilin-mediated trafficking.	(82)

Supplementary Table 3.Evidence level and translational barriers of natural products and traditional Chinese medicine formulas proposed for microglial state calibration in Alzheimer's disease

Note: Translational certainty ratings reflect the overall strength of human evidence, reproducibility, and feasibility for clinical development. AD, Alzheimer's disease; AMPK, AMP-activated protein kinase; BBB, blood–brain barrier; CNS, central nervous system; CSF, cerebrospinal fluid;

CYP, cytochrome P450; GMP, good manufacturing practice; LDAM, lipid droplet-accumulating microglia; mTOR, mechanistic target of rapamycin; NF-κB, nuclear factor kappa B; NLRP3, NOD-like receptor family pyrin domain containing 3; PD, pharmacodynamics; PI3K, phosphoinositide 3-kinase; PK, pharmacokinetics; *SIRT1*, sirtuin 1; TCM, traditional Chinese medicine; TREM2, triggering receptor expressed on myeloid cells 2.

Evidence level and translational barriers of natural products and traditional Chinese medicine formulas proposed for microglial state calibration in Alzheimer's disease

**Agent / formula**	**Main proposed active constituents or pharmacological class**	**Current AD-related evidence base**	**Main microglia-related rationale**	**CNS pharmacokinetic / bioavailability concern**	**Standardization and batch-variability concern**	**Potential herb-drug or drug-drug interaction concern**	**Human AD clinical evidence**	**Regulatory / methodological barrier**	**Translational certainty**	**Reference(s)**
Curcumin / nano-curcumin	Polyphenolic diarylheptanoid; anti-inflammatory and antioxidant compound	Preclinical AD models; nanotheranostic and delivery studies; limited human cognitive studies outside robust disease-modifying settings	May reduce NF-κB/*NLRP3*-related inflammatory signaling, oxidative stress, and microglial overactivation; may influence amyloid-associated inflammatory microenvironment	Native curcumin has very low oral bioavailability, rapid metabolism, poor aqueous solubility, and uncertain	Purity, formulation, particle size, excipients, and curcuminoid composition vary substantially across	Possible interaction with anticoagulants, antiplatelet agents, CYP enzymes, and P-glycoprotein-related transport; interaction	No definitive disease-modifying AD trial evidence; curcumin-based studies remain limited by formulation	Requires validated CNS exposure, standardized formulation, scalable manufacturing, and biomarker	Low to medium	(27–29, 32, 39, 76, 83, 84)

Evidence level and translational barriers of natural products and traditional Chinese medicine formulas proposed for microglial state calibration in Alzheimer’s disease										
**Agent / formula**	**Main proposed active constituents or pharmacological class**	**Current AD-related evidence base**	**Main microglia-related rationale**	**CNS pharmacokinetic / bioavailability concern**	**Standardization and batch-variability concern**	**Potential herb-drug or drug-drug interaction concern**	**Human AD clinical evidence**	**Regulatory / methodological barrier**	**Translational certainty**	**Reference(s)**
				human brain exposure	preparations	risk may increase in elderly patients receiving polypharmacy	n and exposure issues	-linked proof of microglial target engagement		
Berberine / berberine-containing combinations	Isoquinoline alkaloid with metabolic, anti-inflammatory, and AMPK-related activities	Mainly preclinical AD models; some studies use combination with curcumin or other agents	May modulate AMPK/NF-κB signaling, oxidative stress, and inflammatory microglial activation; may influence insulin-resistance-like pathways	Oral bioavailability is low; extensive first-pass metabolism; BBB penetration and active	Source species, extraction process, alkaloid content, and contaminant control require	Potential interaction with CYP enzymes, P-glycoprotein, hypoglycemic drugs, antihypertensive agents,	No convincing complete d AD clinical trial evidence for berberine	Requires single-agent versus combination clarification, PK/PD modeling, CNS exposure	Low	(27–29, 85)

Evidence level and translational barriers of natural products and traditional Chinese medicine formulas proposed for microglial state calibration in Alzheimer's disease										
**Agent / formula**	**Main proposed active constituents or pharmacological class**	**Current AD-related evidence base**	**Main microglia-related rationale**	**CNS pharmacokinetic / bioavailability concern**	**Standardization and batch-variability concern**	**Potential herb-drug or drug-drug interaction concern**	**Human AD clinical evidence**	**Regulatory / methodological barrier**	**Translational certainty**	**Reference(s)**
			relevant to AD	CNS concentrations remain uncertain	strict standardization	and anticoagulants	as a disease-modifying therapy	confirmation, and safety testing in elderly AD populations		
Resveratrol	Stilbene polyphenol; SIRT1-linked metabolic and anti-inflammatory modulator	Preclinical AD models and phase II human AD trial evidence for selected biomarker changes	May regulate *SIRT1*-related pathways, mitochondrial quality control, inflammatory signaling, and proteostasis; may reduce selected inflammatory	Rapid metabolism and low systemic bioavailability; CNS exposure depends on dose, formulatio	Commercial preparations differ in trans-resveratrol content, stability, and impurity	May interact with anticoagulants, antiplatelet drugs, CYP enzymes, and high-dose polyphenol supplementat	Phase II AD trial reported biomarker changes such as CSF MMP9 and Aβ40 alteration	Requires replication, optimized formulation, biomarker-defined responder selection,	Medium	(77, 78, 86)

Evidence level and translational barriers of natural products and traditional Chinese medicine formulas proposed for microglial state calibration in Alzheimer's disease										
**Agent / formula**	**Main proposed active constituents or pharmacolog ical class**	**Current AD-related evidence base**	**Main microglia-related rationale**	**CNS pharmac okinetic / bioavaila bility concern**	**Standardi zation and batch-var iability concern**	**Potential herb-drug or drug-drug interaction concern**	**Human AD clinical evidence**	**Regulato ry / methodol ogical barrier**	**Transl ational certain ty**	**Refere nce(s)**
			biomarkers	n, and metabolit es	profile	ion	s, but cognitive efficacy remains inconclusi ve	and clear linkage to microglial state endpoints		
Liuwei Dihuang Pill	Multi-herb traditional formula; contains multiple iridoids, phenolics, polysaccharid es, and other constituents depending on	Preclinical APP/PS1 mouse evidence; no established AD clinical efficacy trial	May rebalance PI3K/Akt-associat ed survival signaling, inflammatory mediators, and glial activation in animal models	CNS-activ e constitue nts and effective human brain concentra tions are not well defined	High complexit y; herb source, processin g, extraction method, dose equivalen ce, and	Potential interaction with antidiabetic, antihypertens ive, anticoagulant , or cholinesteras e-inhibiting drugs cannot	No definitive AD populatio n clinical trial evidence for disease modificati on	Requires GMP-leve l standardi zation, chemical fingerprint ing, batch release criteria, toxicology	Low	(30)

Evidence level and translational barriers of natural products and traditional Chinese medicine formulas proposed for microglial state calibration in Alzheimer's disease										
**Agent / formula**	**Main proposed active constituents or pharmacological class**	**Current AD-related evidence base**	**Main microglia-related rationale**	**CNS pharmacokinetic / bioavailability concern**	**Standardization and batch-variability concern**	**Potential herb-drug or drug-drug interaction concern**	**Human AD clinical evidence**	**Regulatory / methodological barrier**	**Translational certainty**	**Reference(s)**
	preparation				marker-compound quantification are essential	be excluded without systematic testing		, and clinically meaningful endpoints		
Yishen Huazhuo decoction	Multi-component TCM decoction; composition-dependent mixture with putative immunometabolic activity	Preclinical APP/PS1 mouse evidence	May reshape TREM2/NF-κB signaling, promote phagocytic/resolution-associated microglial phenotype, reduce plaque burden, and improve working memory in mouse models	Human CNS pharmacokinetics of active constituents are unknown; decoction-derived metabolites may	Decoction preparation, herb origin, extraction time, concentration, and storage conditions may strongly	Multi-component formula creates risk of pharmacological noise and unpredictable interaction with anti-amyloid antibodies, anticoagulant	No completed AD clinical trial evidence demonstrating efficacy	Requires active-constituent identification, quality-control markers, interaction studies, and staged	Low	(31)

Evidence level and translational barriers of natural products and traditional Chinese medicine formulas proposed for microglial state calibration in Alzheimer's disease										
**Agent / formula**	**Main proposed active constituents or pharmacological class**	**Current AD-related evidence base**	**Main microglia-related rationale**	**CNS pharmacokinetic / bioavailability concern**	**Standardization and batch-variability concern**	**Potential herb-drug or drug-drug interaction concern**	**Human AD clinical evidence**	**Regulatory / methodological barrier**	**Translational certainty**	**Reference(s)**
				differ from parent compounds	affect composition	s, antidiabetic drugs, or anti-inflammatory agents		clinical development before combination with monoclonal antibodies		
Cycloastragenol	Triterpenoid saponin-related compound derived from Astragalus species	Preclinical and network pharmacology-supported AD model evidence	Proposed to target microglia-related inflammatory networks and aging-associated pathways; may reduce neuroinflammation in experimental	BBB penetration, active CNS concentration, and human PK/PD relationsh	Purity, source plant, extraction process, and metabolite profile require	Potential interaction with immunomodulatory agents and metabolic drugs remains	No definitive AD clinical efficacy trial evidence	Requires mechanistic confirmation in human microglia, dose-response	Low	(87)

Evidence level and translational barriers of natural products and traditional Chinese medicine formulas proposed for microglial state calibration in Alzheimer's disease										
**Agent / formula**	**Main proposed active constituents or pharmacological class**	**Current AD-related evidence base**	**Main microglia-related rationale**	**CNS pharmacokinetic / bioavailability concern**	**Standardization and batch-variability concern**	**Potential herb-drug or drug-drug interaction concern**	**Human AD clinical evidence**	**Regulatory / methodological barrier**	**Translational certainty**	**Reference(s)**
			models	ip remain insufficiently established	validation	insufficiently characterized		studies, CNS exposure validation, and safety assessment		
Berbamine	Bisbenzylisoquinoline alkaloid; proposed mTOR/autophagy-related modulator	Preclinical AD model evidence if supported by cited study	May influence autophagic-lysosomal flux and amyloid clearance through mTOR-related mechanisms	CNS exposure and therapeutic window in humans are unclear	Alkaloid purity and source standardization required	Potential kinase-related off-target effects and interaction with autophagy-modulating drugs require evaluation	No established AD clinical trial evidence	Requires confirmation of target specificity, lysosomal safety, and risk of autophagi	Low	(88)

Evidence level and translational barriers of natural products and traditional Chinese medicine formulas proposed for microglial state calibration in Alzheimer's disease										
**Agent / formula**	**Main proposed active constituents or pharmacological class**	**Current AD-related evidence base**	**Main microglia-related rationale**	**CNS pharmacokinetic / bioavailability concern**	**Standardization and batch-variability concern**	**Potential herb-drug or drug-drug interaction concern**	**Human AD clinical evidence**	**Regulatory / methodological barrier**	**Translational certainty**	**Reference(s)**
								c stress in LDAM-like microglia		
Multi-component TCM formulas as a class	Dozens to hundreds of parent compounds and metabolites; network pharmacology often predicts multi-target activity	Mostly preclinical or computational evidence	May theoretically act across inflammation, lipid metabolism, oxidative stress, autophagy, and vascular dysfunction	Active CNS-exposed constituents are usually unidentified; plasma exposure does not prove brain	High batch variability unless controlled by chemical fingerprinting, quantitative marker compounds, and	Pharmacological noise is a major concern; multiple constituents may have synergistic, antagonistic, or toxic interactions	Formula-specific AD clinical evidence is generally absent or insufficient	Requires deconvolution of active fractions, reproducible manufacturing, PK/PD integration, interactio	Low	(27–29, 89–93)

Evidence level and translational barriers of natural products and traditional Chinese medicine formulas proposed for microglial state calibration in Alzheimer's disease										
**Agent / formula**	**Main proposed active constituents or pharmacological class**	**Current AD-related evidence base**	**Main microglia-related rationale**	**CNS pharmacokinetic / bioavailability concern**	**Standardization and batch-variability concern**	**Potential herb-drug or drug-drug interaction concern**	**Human AD clinical evidence**	**Regulatory / methodological barrier**	**Translational certainty**	**Reference(s)**
				target engagement	GMP manufacturing			n studies, and biomarker-defined clinical trials		

References

1. Bernier L-P, York EM, MacVicar BA. Immunometabolism in the brain: how metabolism shapes microglial function. Trends Neurosci. (2020) 43:854–870. doi:10.1016/j.tins.2020.08.008
2. Lu N, Jin Z, Liu N, Zhu C, Wei H, Xu Q. Microglial glycolytic reprogramming in Alzheimer's disease: association with impaired phagocytic function and altered vascular proximity. J Neuroinflammation. (2025) 22:223. doi:10.1186/s12974-025-03546-9
3. Jung ES, Choi H, Mook-Jung I. Decoding microglial immunometabolism: a new frontier in Alzheimer's disease research. Mol Neurodegener. (2025) 20:37. doi:10.1186/s13024-025-00825-0

4. Wu Q-L, Yang X, Luo J-X, Liu L, Zhou Y, Lu M-H. Microglia energy metabolism: a new perspective on Alzheimer's disease treatment. J Neurol Sci. (2025) 475:123585. doi:10.1016/j.jns.2025.123585
5. Tannahill GM, Curtis AM, Adamik J, Palsson-McDermott EM, McGettrick AF, Goel G, et al. Succinate is an inflammatory signal that induces IL-1β through HIF-1α. Nature. (2013) 496:238–242. doi:10.1038/nature11986
6. Palsson-McDermott EM, Curtis AM, Goel G, Lauterbach MAR, Sheedy FJ, Gleeson LE, et al. Pyruvate kinase M2 regulates HIF-1α activity and IL-1β induction and is a critical determinant of the Warburg effect in LPS-activated macrophages. Cell Metab. (2015) 21:65–80. doi:10.1016/j.cmet.2014.12.005
7. Shokr MM. Rewiring brain immunity: targeting microglial metabolism for neuroprotection in neurodegenerative disorders. Metab Brain Dis. (2025) 40:326. doi:10.1007/s11011-025-01739-y
8. Noh M-Y, Kwon HS, Kwon M-S, Nahm M, Jin HK, Bae J, et al. Biomarkers and therapeutic strategies targeting microglia in neurodegenerative diseases: current status and future directions. Mol Neurodegener. (2025) 20:82. doi:10.1186/s13024-025-00867-4
9. Millet A, Ledo JH, Tavazoie SF. An exhausted-like microglial population accumulates in aged and APOE4 genotype Alzheimer's brains. Immunity. (2024) 57:153–170.e6. doi:10.1016/j.immuni.2023.12.001
10. Marschallinger J, Iram T, Zardeneta M, Lee SE, Lehallier B, Haney MS, et al. Lipid-droplet-accumulating microglia represent a dysfunctional and proinflammatory state in the aging brain. Nat Neurosci. (2020) 23:194–204. doi:10.1038/s41593-019-0566-1
11. Li Y, Xia X, Wang Y, Zheng JC. Mitochondrial dysfunction in microglia: a novel perspective for pathogenesis of Alzheimer's disease. J Neuroinflammation. (2022) 19:248. doi:10.1186/s12974-022-02613-9
12. Rahman MA, Rahman MDH, Rhim H, Kim B. Drug target to alleviate mitochondrial dysfunctions in Alzheimer's disease: recent advances and therapeutic implications. Curr Neuropharmacol. (2024) 22:1942–1959. doi:10.2174/1570159X22666240426091311
13. Singh A, Kumar A. Microglial inhibitory mechanism of coenzyme Q10 against Aβ(1–42)-induced cognitive dysfunctions: possible behavioral, biochemical, cellular, and histopathological alterations. Front Pharmacol. (2015) 6. doi: 10.3389/fphar.2015.00268
14. Fišar Z, Hroudová J. CoQ10 and mitochondrial dysfunction in Alzheimer's disease. Antioxidants. (2024) 13:191. doi: 10.3390/antiox13020191
15. West AP, Khoury-Hanold W, Staron M, Tal MC, Pineda CM, Lang SM, et al. Mitochondrial DNA stress primes the antiviral innate immune response. Nature. (2015) 520:553–557. doi:10.1038/nature14156

16. White MJ, McArthur K, Metcalf D, Lane RM, Cambier JC, Herold MJ, et al. Apoptotic caspases suppress mtDNA-induced STING-mediated type I IFN production. Cell. (2014) 159:1549–1562. doi:10.1016/j.cell.2014.11.036
17. McArthur K, Whitehead LW, Heddleston JM, Li L, Padman BS, Oorschot V, et al. BAK/BAX macropores facilitate mitochondrial herniation and mtDNA efflux during apoptosis. Science. (2018) 359:eaao6047. doi:10.1126/science.aao6047
18. Decout A, Katz JD, Venkatraman S, Ablasser A. The cGAS–STING pathway as a therapeutic target in inflammatory diseases. Nat Rev Immunol. (2021) 21:548–569. doi:10.1038/s41577-021-00524-z
19. Mary A, Barale S, Eysert F, Valverde A, Lacas-Gervais S, Bauer C, et al. Hampered AMPK-ULK1 cascade in Alzheimer's disease instigates mitochondria dysfunctions and AD-related alterations which are alleviated by metformin. Alzheimers Res Ther. (2025) 17:127. doi:10.1186/s13195-025-01772-0
20. Quick JD, Silva C, Wong JH, Lim KL, Reynolds R, Barron AM, et al. Lysosomal acidification dysfunction in microglia: an emerging pathogenic mechanism of neuroinflammation and neurodegeneration. J Neuroinflammation. (2023) 20:185. doi:10.1186/s12974-023-02866-y
21. Nixon RA. The role of autophagy in neurodegenerative disease. Nat Med. (2013) 19:983–997. doi:10.1038/nm.3232
22. Menzies FM, Fleming A, Rubinsztein DC. Compromised autophagy and neurodegenerative diseases. Nat Rev Neurosci. (2015) 16:345–357. doi:10.1038/nrn3961
23. Kim J, Kundu M, Viollet B, Guan K-L. AMPK and mTOR regulate autophagy through direct phosphorylation of ULK1. Nat Cell Biol. (2011) 13:132–141. doi:10.1038/ncb2152
24. Egan DF, Shackelford DB, Mihaylova MM, Gelino S, Kohnz RA, Mair W, et al. Phosphorylation of ULK1 by AMP-activated protein kinase connects energy sensing to mitophagy. Science. (2011) 331:456–461. doi:10.1126/science.1196371
25. Gwinn DM, Shackelford DB, Egan DF, Mihaylova MM, Mery A, Vasquez DS, et al. AMPK phosphorylation of Raptor mediates a metabolic checkpoint. Mol Cell. (2008) 30:214–226. doi:10.1016/j.molcel.2008.03.003
26. Gonzales MM, Garbarino VR, Kautz TF, Song X, Lopez-Cruzan M, Linehan L, et al. Rapamycin treatment for Alzheimer's disease and related dementias: a pilot phase 1 clinical trial. Commun Med. (2025) 5:189. doi: 10.1038/s43856-025-00904-9
27. Bhattacharya RS, Singh R, Panghal A, Thakur A, Singh L, Verma RK, et al. Multi-targeting phytochemicals for Alzheimer's disease. Phytother Res. (2025) 39:1453–1483. doi: 10.1002/ptr.8435

28. Shen Y, Liu F, Zhang M. Therapeutic potential of plant-derived natural compounds in Alzheimer's disease: targeting microglia-mediated neuroinflammation. Biomed Pharmacother. (2024) 178:117235. doi: 10.1016/j.biopha.2024.117235
29. Huang J, Huang N, Mao Q, Shi J, Qiu Y. Natural bioactive compounds in Alzheimer's disease: from the perspective of type 3 diabetes mellitus. Front Aging Neurosci. (2023) 15:1130253. doi: 10.3389/fnagi.2023.1130253
30. Yuan Y, Liu Y, Hao L, Ma J, Shao S, Yu Z, et al. The neuroprotective effects of Liuwei Dihuang medicine in the APP/PS1 mouse model are dependent on the PI3K/Akt signaling pathway. Front Pharmacol. (2023) 14:1188893. doi: 10.3389/fphar.2023.1188893
31. Wang K, Zan S, Xu J, Sun W, Li C, Zhang W, et al. Yishen Huazhuo decoction regulates microglial polarization to reduce Alzheimer's disease-related neuroinflammation through TREM2. Heliyon. (2024) 10:e35800. doi: 10.1016/j.heliyon.2024.e35800
32. Shabbir U, Rubab M, Tyagi A, Oh D-H. Curcumin and its derivatives as theranostic agents in Alzheimer's disease: the implication of nanotechnology. Int J Mol Sci. (2020) 22:196. doi: 10.3390/ijms22010196
33. Chen L, Guan Y, Wang S, Han X, Guo F, Wang Y. Engineered nanoplatforms for brain-targeted co-delivery of phytochemicals in Alzheimer's disease: rational design, blood-brain barrier penetration, and multi-target therapeutic synergy. Neurotherapeutics. (2025) 22:e00722. doi: 10.1016/j.neurot.2025.e00722
34. Gao L, Wang J, Bi Y. Nanotechnology for neurodegenerative diseases: recent progress in brain-targeted delivery, stimuli-responsive platforms, and organelle-specific therapeutics. Int J Nanomedicine. (2025) 20:11015–11044. doi: 10.2147/IJN.S549893
35. Liu N, Ruan J, Li H, Fu J. Nanoparticles loaded with natural medicines for the treatment of Alzheimer's disease. Front Neurosci. (2023) 17:1112435. doi: 10.3389/fnins.2023.1112435
36. Zhou P, Chao Q, Li C, Wang N, Guo S, Wang P, et al. Microglia-targeting nanosystems that cooperatively deliver Chinese herbal ingredients alleviate behavioral and cognitive deficits in Alzheimer's disease model mice. J Nanobiotechnol. (2025) 23:313. doi: 10.1186/s12951-025-03385-z
37. Liao J, Fan L, Li Y, Xu Q-Q, Xiong L-Y, Zhang S-S, et al. Recent advances in biomimetic nanodelivery systems: new brain-targeting strategies. J Control Release. (2023) 358:439–464. doi: 10.1016/j.jconrel.2023.05.009
38. Gao X, Xu J, Yao T, Liu X, Zhang H, Zhan C. Peptide-decorated nanocarriers penetrating the blood-brain barrier for imaging and therapy of brain diseases. Adv Drug Deliv Rev. (2022) 187:114362. doi: 10.1016/j.addr.2022.114362

39. Ruan Y, Xiong Y, Fang W, Yu Q, Mai Y, Cao Z, et al. Highly sensitive curcumin-conjugated nanotheranostic platform for detecting amyloid-beta plaques by magnetic resonance imaging and reversing cognitive deficits of Alzheimer's disease via NLRP3 inhibition. J Nanobiotechnol. (2022) 20:322. doi: 10.1186/s12951-022-01524-4
40. van Dyck CH, Swanson CJ, Aisen P, Bateman RJ, Chen C, Gee M, et al. Lecanemab in early Alzheimer's disease. N Engl J Med. (2023) 388:9–21. doi:10.1056/NEJMoa2212948
41. Sims JR, Zimmer JA, Evans CD, Lu M, Ardayfio P, Sparks J, et al. Donanemab in early symptomatic Alzheimer disease: the TRAILBLAZER-ALZ 2 randomized clinical trial. JAMA. (2023) 330:512–527. doi:10.1001/jama.2023.13239
42. Avgerinos KI, Manolopoulos A, Ferrucci L, Kapogiannis D. Critical assessment of anti-amyloid-β monoclonal antibodies effects in Alzheimer's disease: a systematic review and meta-analysis highlighting target engagement and clinical meaningfulness. Sci Rep. (2024) 14:25741. doi:10.1038/s41598-024-75204-8
43. Sperling RA, Jack CR, Black SE, Frosch MP, Greenberg SM, Hyman BT, et al. Amyloid-related imaging abnormalities in amyloid-modifying therapeutic trials: recommendations from the Alzheimer's Association Research Roundtable Workgroup. Alzheimers Dement. (2011) 7:367–385. doi: 10.1016/j.jalz.2011.05.2351
44. Faraco G, Park L, Anrather J, Iadecola C. Brain perivascular macrophages: characterization and functional roles in health and disease. J Mol Med. (2017) 95:1143–1152. doi: 10.1007/s00109-017-1573-f
45. Jordão MJC, Sankowski R, Brendecke SM, Sagar, Locatelli G, Tai Y-H, et al. Single-cell profiling identifies myeloid cell subsets with distinct fates during neuroinflammation. Science. (2019) 363:eaat7554. doi: 10.1126/science.aat7554
46. Arnold SE, Hyman BT, Betensky RA, Dodge HH. Pathways to personalized medicine—embracing heterogeneity for progress in clinical therapeutics research in Alzheimer's disease. Alzheimers Dement. (2024) 20:7384–7394. doi:10.1002/alz.14063
47. Franzmeier N, Suárez-Calvet M, Frontzkowski L, Moore A, Hohman TJ, Morenas-Rodriguez E, et al. Higher CSF sTREM2 attenuates ApoE4-related risk for cognitive decline and neurodegeneration. Mol Neurodegener. (2020) 15:57. doi:10.1186/s13024-020-00407-2
48. Chen M-K, Mecca AP, Naganawa M, Finnema SJ, Toyonaga T, Lin S, et al. Assessing synaptic density in Alzheimer disease with synaptic vesicle glycoprotein 2A positron emission tomographic imaging. JAMA Neurol. (2018) 75:1215–1224. doi: 10.1001/jamaneurol.2018.1836
49. Long H, Simmons A, Mayorga A, Burgess B, Nguyen T, Budda B, et al. Preclinical and first-in-human evaluation of AL002, a novel TREM2 agonistic antibody for Alzheimer's disease. Alzheimers Res Ther. (2024) 16:235. doi:10.1186/s13195-024-01599-1

50. ClinicalTrials.gov. A Phase I Study for Safety and Tolerability of AL002. Identifier NCT03635047. Available at: https://clinicaltrials.gov/study/NCT03635047 [Accessed March 30, 2026].
51. ClinicalTrials.gov. A Phase 2 Study to Evaluate Efficacy and Safety of AL002 in Participants With Early Alzheimer's Disease. Identifier NCT04592874. Available at: https://clinicaltrials.gov/study/NCT04592874 [Accessed March 30, 2026].
52. ClinicalTrials.gov. A Long-term Extension Study to Evaluate Safety, Tolerability, and Efficacy of AL002 in Alzheimer's Disease. Identifier NCT05744401. Available at: https://clinicaltrials.gov/study/NCT05744401 [Accessed March 30, 2026].
53. ClinicalTrials.gov. A Phase 1 Study of VG-3927 in Healthy Adults and Patients With Alzheimer's Disease. Identifier NCT06343636. Available at: https://clinicaltrials.gov/study/NCT06343636 [Accessed March 30, 2026].
54. ClinicalTrials.gov. First in Human Study for Safety and Tolerability of AL003. Identifier NCT03822208. Available at: https://clinicaltrials.gov/study/NCT03822208 [Accessed March 30, 2026].
55. Koenig AM, Mechanic-Hamilton D, Xie SX, Combs MF, Cappola AR, Xie L, et al. Effects of the insulin sensitizer metformin in Alzheimer disease: pilot data from a randomized placebo-controlled crossover study. Alzheimer Dis Assoc Disord. (2017) 31:107–113. doi:10.1097/WAD.0000000000000202
56. ClinicalTrials.gov. Effect of Insulin Sensitizer Metformin on AD Biomarkers. Identifier NCT01965756. Available at: https://clinicaltrials.gov/study/NCT01965756 [Accessed March 30, 2026].
57. ClinicalTrials.gov. Metformin in Alzheimer's Dementia Prevention. Identifier NCT04098666. Available at: https://clinicaltrials.gov/study/NCT04098666 [Accessed March 30, 2026].
58. ClinicalTrials.gov. Gene Therapy for APOE4 Homozygote of Alzheimer's Disease. Identifier NCT03634007. Available at: https://clinicaltrials.gov/study/NCT03634007 [Accessed March 30, 2026].
59. ClinicalTrials.gov. Safety and Pharmacokinetics of Single Ascending Doses and Multiple Ascending Doses of CS6253 in Healthy Volunteers. Identifier NCT05965414. Available at: https://clinicaltrials.gov/study/NCT05965414 [Accessed March 30, 2026].
60. ClinicalTrials.gov. Cognition, Age, and RaPamycin Effectiveness - DownregulatIon of thE mTor Pathway. Identifier NCT04200911. Available at: https://clinicaltrials.gov/study/NCT04200911 [Accessed March 30, 2026].
61. ClinicalTrials.gov. Rapamycin - Effects on Alzheimer's and Cognitive Health. Identifier NCT04629495. Available at: https://clinicaltrials.gov/study/NCT04629495 [Accessed March 30, 2026].

62. ClinicalTrials.gov. Evaluating the Efficacy and Safety of Nilotinib BE in Subjects With Early Alzheimer's Disease. Identifier NCT05143528. Available at: https://clinicaltrials.gov/study/NCT05143528 [Accessed March 30, 2026].
63. ClinicalTrials.gov. A Study to Evaluate the Safety, Tolerability and Amount of EI-1071 in Blood in Healthy Volunteers. Identifier NCT04238364. Available at: https://clinicaltrials.gov/study/NCT04238364 [Accessed March 30, 2026].
64. ClinicalTrials.gov. Masitinib in Patients With Mild Alzheimer's Disease. Identifier NCT05564169. Available at: https://clinicaltrials.gov/study/NCT05564169 [Accessed March 30, 2026].
65. Potter H, Woodcock JH, Boyd TD, Coughlan CM, O'Shaughnessy JR, Borges MT, et al. Safety and efficacy of sargramostim in the treatment of Alzheimer's disease. Alzheimers Dement (N Y). (2021) 7:e12158. doi: 10.1002/trc2.12158
66. ClinicalTrials.gov. Study of the Safety & Efficacy of Leukine® in the Treatment of Alzheimer's Disease. Identifier NCT01409915. Available at: https://clinicaltrials.gov/study/NCT01409915 [Accessed March 30, 2026].
67. ClinicalTrials.gov. Phase II Trial to Evaluate Safety and Efficacy of GM-CSF/Sargramostim in Alzheimer's Disease. Identifier NCT04902703. Available at: https://clinicaltrials.gov/study/NCT04902703 [Accessed March 30, 2026].
68. Gordon ML, Christen E, Keehlisen L, Gong M, Lam F, Giliberto L, et al. An open-label, pilot study of daratumumab SC in mild to moderate Alzheimer's disease. J Alzheimers Dis Rep. (2024) 8:1111–1114. doi: 10.3233/ADR-240089
69. ClinicalTrials.gov. Study of Daratumumab in Patients With Mild to Moderate Alzheimer's Disease. Identifier NCT04070378. Available at: https://clinicaltrials.gov/study/NCT04070378 [Accessed March 30, 2026].
70. ClinicalTrials.gov. Study of the Efficacy and Safety of Various Anti-inflammatory Agents in Participants With Mild Cognitive Impairment or Mild Alzheimer's Disease. Identifier NCT04795466. Available at: https://clinicaltrials.gov/study/NCT04795466 [Accessed March 30, 2026].
71. ClinicalTrials.gov. Neurodegenerative Alzheimer's Disease and Amyotrophic Lateral Sclerosis Basket Trial. Identifier NCT05189106. Available at: https://clinicaltrials.gov/study/NCT05189106 [Accessed March 30, 2026].
72. ClinicalTrials.gov. A Research Study Investigating Semaglutide in People With Early Alzheimer's Disease. Identifier NCT04777396. Available at: https://clinicaltrials.gov/study/NCT04777396 [Accessed March 30, 2026].
73. ClinicalTrials.gov. Study to Assess the Safety, Tolerability, Pharmacokinetics, Pharmacodynamics, and Efficacy of TB006 in Participants With Alzheimer's Disease. Identifier NCT05074498. Available at: https://clinicaltrials.gov/study/NCT05074498 [Accessed March 30, 2026].

74. ClinicalTrials.gov. A Long Term Extension Study to Assess the Safety of TB006 in Participants With Alzheimer's Disease. Identifier NCT05476783. Available at: https://clinicaltrials.gov/study/NCT05476783 [Accessed March 30, 2026].
75. ClinicalTrials.gov. A Trial Evaluating the Effect of NIO752 on Tau Synthesis Measured by a Process Known as SILK. Identifier NCT06372821. Available at: https://clinicaltrials.gov/study/NCT06372821 [Accessed March 30, 2026].
76. ClinicalTrials.gov. Curcumin and Yoga Therapy for Those at Risk for Alzheimer's Disease. Identifier NCT01811381. Available at: https://clinicaltrials.gov/study/NCT01811381 [Accessed March 30, 2026].
77. Turner RS, Thomas RG, Craft S, van Dyck CH, Mintzer J, Reynolds BA, et al. A randomized, double-blind, placebo-controlled trial of resveratrol for Alzheimer disease. Neurology. (2015) 85:1383–1391. doi: 10.1212/WNL.0000000000002035
78. ClinicalTrials.gov. Resveratrol for Alzheimer's Disease. Identifier NCT01504854. Available at: https://clinicaltrials.gov/study/NCT01504854 [Accessed March 30, 2026].
79. ClinicalTrials.gov. Randomized Trial of a Nutritional Supplement in Alzheimer's Disease. Identifier NCT00678431. Available at: https://clinicaltrials.gov/study/NCT00678431 [Accessed March 30, 2026].
80. ClinicalTrials.gov. Mycose AdminiStration for HealIng Alzheimer NEuropathy. Identifier NCT04663854. Available at: https://clinicaltrials.gov/study/NCT04663854 [Accessed March 30, 2026].
81. ClinicalTrials.gov. Safety and Efficacy Evaluation of Allogenic Adipose MSC-Exos in Patients With Alzheimer's Disease. Identifier NCT04388982. Available at: https://clinicaltrials.gov/study/NCT04388982 [Accessed March 30, 2026].
82. ClinicalTrials.gov. Efficacy and Safety of GSK4527226 [AL101] in Participants With Early Alzheimer's Disease. Identifier NCT06079190. Available at: https://clinicaltrials.gov/study/NCT06079190 [Accessed March 30, 2026].
83. Heneka MT, Kummer MP, Stutz A, Delekate A, Schwartz S, Vieira-Saecker A, et al. NLRP3 is activated in Alzheimer's disease and contributes to pathology in APP/PS1 mice. Nature. (2013) 493:674–678. doi: 10.1038/nature11729
84. Shao S, Ye X, Su W, Wang Y. Curcumin alleviates Alzheimer's disease by inhibiting inflammatory response, oxidative stress and activating the AMPK pathway. J Chem Neuroanat. (2023) 134:102363. doi: 10.1016/j.jchemneu.2023.102363
85. Durairajan SSK, Liu L-F, Lu J-H, Chen L-L, Yuan Q, Chung SK, et al. Berberine ameliorates β-amyloid pathology, gliosis, and cognitive impairment in an Alzheimer's disease transgenic mouse model. Neurobiol Aging. (2012) 33:2903–2919. doi: 10.1016/j.neurobiolaging.2012.02.016

86. Gureev A, Sadovnikova I, Chernyshova E, Krutskikh E, Pevzner I, Zorova L, et al. Resveratrol preserves mitochondrial DNA integrity and long-term memory without decreasing amyloid-β levels in Alzheimer's disease mouse models. BIOCELL. (2025) 49:873–892. doi: 10.32604/biocell.2025.063557
87. Weng W, Lin B, Zheng J, Sun Y, Li Z, Chen X, et al. Novel application of cycloastragenol target microglia for the treatment of Alzheimer's disease: evidence from single-cell analysis, network pharmacology and experimental assessment. Phytomedicine. (2025) 139:156502. doi: 10.1016/j.phymed.2025.156502
88. Ge P, Guo S, Wang P, Zhou P, Tang Z, Yang N, et al. Berbamine targets the FKBP12-rapamycin-binding domain of the mTOR complex to promote microglial autophagy and ameliorate neuroinflammation in Alzheimer's disease. Phytomedicine. (2025) 142:156771. doi: 10.1016/j.phymed.2025.156771
89. Cummings JL, Osse AML, Kinney JW, Cammann D, Chen J. Alzheimer's disease: combination therapies and clinical trials for combination therapy development. CNS Drugs. (2024) 38:613–624. doi:10.1007/s40263-024-01103-1
90. Bajinka O, Jallow L, Ozdemir G. A multi-target therapeutic framework for Alzheimer's disease: an integrative mechanistic review. Neuroscience. (2026) 596:143–157. doi:10.1016/j.neuroscience.2026.01.010
91. Turgutalp B, Kizil C. Multi-target drugs for Alzheimer's disease. Trends Pharmacol Sci. (2024) 45:628–638. doi:10.1016/j.tips.2024.05.005
92. Hossain MS, Hussain MH. Multi-target drug design in Alzheimer's disease treatment: emerging technologies, advantages, challenges, and limitations. Pharmacol Res Perspect. (2025) 13:e70131. doi:10.1002/prp2.70131
93. Muhammad J, Khan A, Ali A, Fang L, Yanjing W, Xu Q, et al. Network pharmacology: exploring the resources and methodologies. Curr Top Med Chem. (2018) 18:949–964. doi:10.2174/1568026618666180330141351
